# Supplementary material for: Imaging inflammation using an activated macrophage probe with Slc18b1 as the activation-selective gating target
Source: Nat Commun. 2019 Mar 7;10:1111. doi: 10.1038/s41467-019-08990-9 (PMC6405920; doi:10.1038/s41467-019-08990-9)
Supplement: Supplementary file 1 — Supplementary Information [file 41467_2019_8990_MOESM1_ESM.pdf]

Supplementary Information for

Imaging inflammation using an activated macrophage probe with Slc18b1 as the activation-selective gating target

Park, *et al.*

Supplementary Information for

Imaging inflammation using an activated macrophage probe with Slc18b1 as the activation-selective gating target

Sung-Jin Park[1,*], Beomsue Kim[1,*], Sejong Choi[2,*], Sivaraman Balasubramaniam[1], Sung-Chan Lee[1], Jung Yeol Lee[3], Heon Seok Kim[2], Jun-Young Kim[1], Jong-Jin Kim[1,4], Yong-An Lee[1], Nam-Young Kang[1,5], Jin-Soo Kim[2,6], & Young-Tae Chang[1,3,4]

[*]These authors contributed equally to this work.

Correspondence to Jin-Soo Kim (jskim01@snu.ac.kr)

or Young-Tae Chang (ytchang@postech.ac.kr)

Contents of this SI file include:

- Supplementary Figure 1 to 33
- Supplementary Methods including information for the AD and ADCA chemical library (Supplementary Figure 23 to 33)

Other Supplementary information for this manuscript include:

- Supplementary Movie 1. Real-time tracking of activating macrophages with CDg16
- Supplementary Data 1. Spectroscopic properties of the AD and ADCA library
- Supplementary Data 2. Human SLC gene list used for CRISPR activation
- Supplementary Data 3. sgRNA Sequences in the SLC-CRISPRa pools
- Supplementary Data 4. LC/MS data of AD library

List of Supplementary Figures:

Supplementary Figure 1. Evaluation of M1 activated Raw264.7 cells.

Supplementary Figure 2. Screening platform to discriminate M1 macrophages.

Supplementary Figure 3. Synthesis scheme of the Acridine library.

Supplementary Figure 4. CDg16 labels CD86 positive M1 macrophages.

Supplementary Figure 5. The pH independent lysosomal staining of CDg16.

Supplementary Figure 6. The staining pattern of CDg16 and acridine orange in control and M1 macrophages.

Supplementary Figure 7. CDg16 stain pattern in mouse microglia, human macrophages and THP-1 cell line.

Supplementary Figure 8. Application of CDg16 for visualizing atherosclerosis.

Supplementary Figure 9. CDg16 bio-distribution in atherosclerosis disease model.

Supplementary Figure 10. Histological evaluation of CDg16 positive cells in atherosclerotic plaque.

Supplementary Figure 11. CDg16 staining in M1 and M2 activated THP-1 cells.

Supplementary Figure 12. Quantitative analysis of CDg16-stained cells in the aorta of $ApoE^{-/-}$.

Supplementary Figure 13. CDg16 specificity to activated macrophages compared to other cell types of aorta.

Supplementary Figure 14. Visualizing inflamed liver using CDg16.

Supplementary Figure 15. Differential staining pattern of CDg16 in live and dead M1 macrophages.

Supplementary Figure 16. Reversible staining of CDg16 by fixation and permeabilization.

Supplementary Figure 17. Inhibition of endocytosis has no effect on the staining of CDg16.

Supplementary Figure 18. Construction of the SLC-CRISPRa screening system.

Supplementary Figure 19. Evaluation of the SLC-CRISPRa screening system.

Supplementary Figure 20. Enrichment of the CDg16 brightly stained SLC-CRISPRa subpopulation.

Supplementary Figure 21. Flow cytometry and FACS gating strategy.

Supplementary Figure 22. The gene expression patterns of Slc18b1/SLC18B1 in activated Raw264.7 and THP1 cells.

Supplementary Methods

Supplementary Figure 23. Synthetic procedure of the AD and ADCA library

Supplementary Figure 24. General structure of the AD and ADCA library.

Supplementary Figure 25. Normalized absorption and emission spectroscopic property of CDg16.

Supplementary Figure 26. LC/MS Chromatogram of CDg16.

Supplementary Figure 27. ^{1}H-NMR of the compound CDg16 (500 MHz, DMSO-d6).

Supplementary Figure 28. ^{13}C-NMR (500 MHz) of the compound CDg16 (126 MHz, DMSO-d6).

Supplementary Figure 29. ^{1}H-^{13}C-COSY of the compound CDg16 (DMSO-d6)

Supplementary Figure 30. Variable temperature ^{1}H-NMR (350 K) of the compound CDg16 (DMSO-d6, 500 MHz).

Supplementary Figure 31. DEPT (135) of the compound CDg16 (DMSO-d6).

Supplementary Figure 32. High Resolution Mass Spectrum of the compound CDg16.

Supplementary Figure 33. The codes for the AD and ADCA library compounds.

Supplementary Results

Supplementary Figure 1

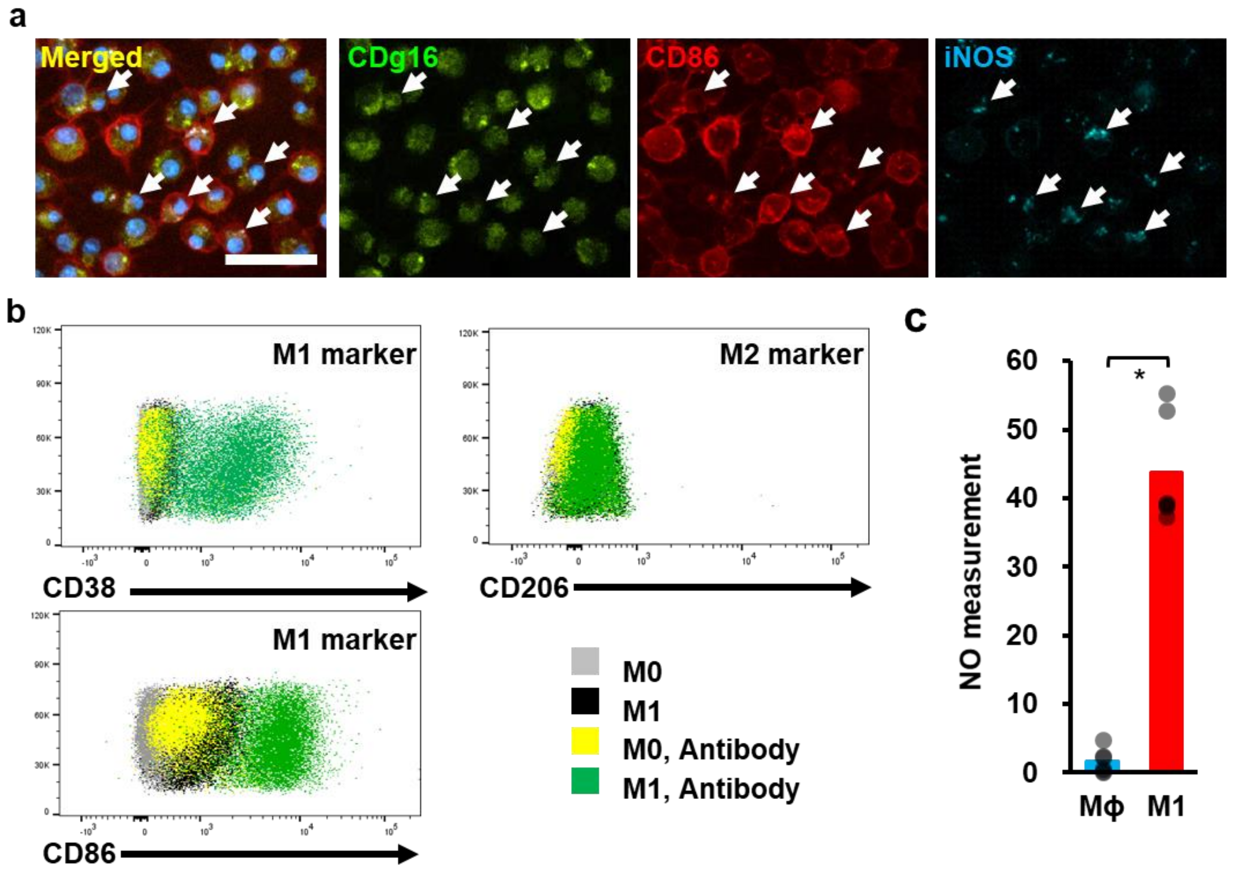

Supplementary Figure 1. Evaluation of M1 activated Raw264.7 cells. Raw264.7 cells were activated to M1 macrophages by incubation 24 hours with lipopolysaccharide (LPS, 100 ng/mL) and interferon-gamma (IFNγ, 20 ng/mL). M1 activation of the CDg16 (1 µM) stained cells were examined by immunocytochemistry with antibodies against CD86 and iNOS (**a**) and by flow cytometry analysis with antibodies against CD38, CD86 for M1, and CD206 for M2 detection (**b**). The amount of nitric oxide production in control and M1 activated Raw264.7 cells were measured using Griess reagent (**c**). The bar graph in (**c**) is presented as mean value with each data point (dots) (N=6 and *, $p < 0.01$).

Supplementary Figure 2.

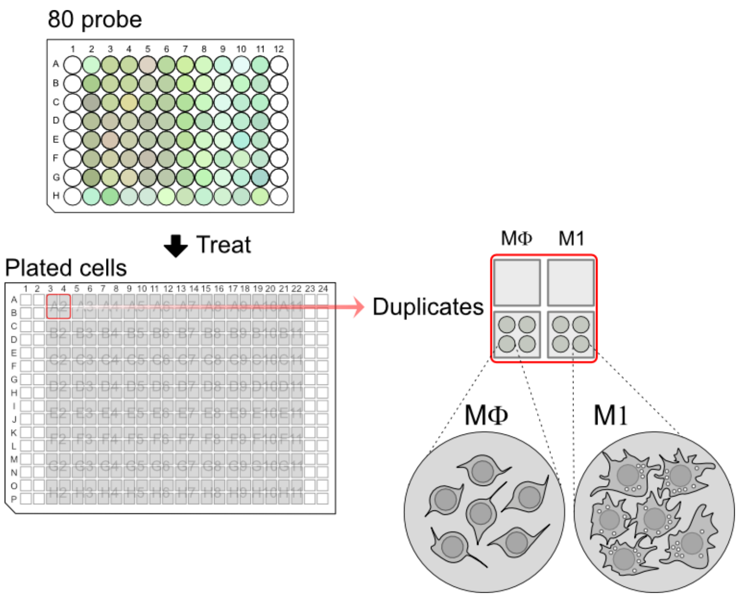

Supplementary Figure 2. Screening platform to discriminate M1 macrophages. Each probe of 80 compounds in a 96-well plate was treated as duplicates to the two types of live cells in a 384-well plate, non-activated (Mϕ) and LPS/IFNγ-activated (M1) Raw264.7 macrophages. The image-based analysis was performed after one-hour incubation with a probe (1 μM).

Supplementary Figure 3.

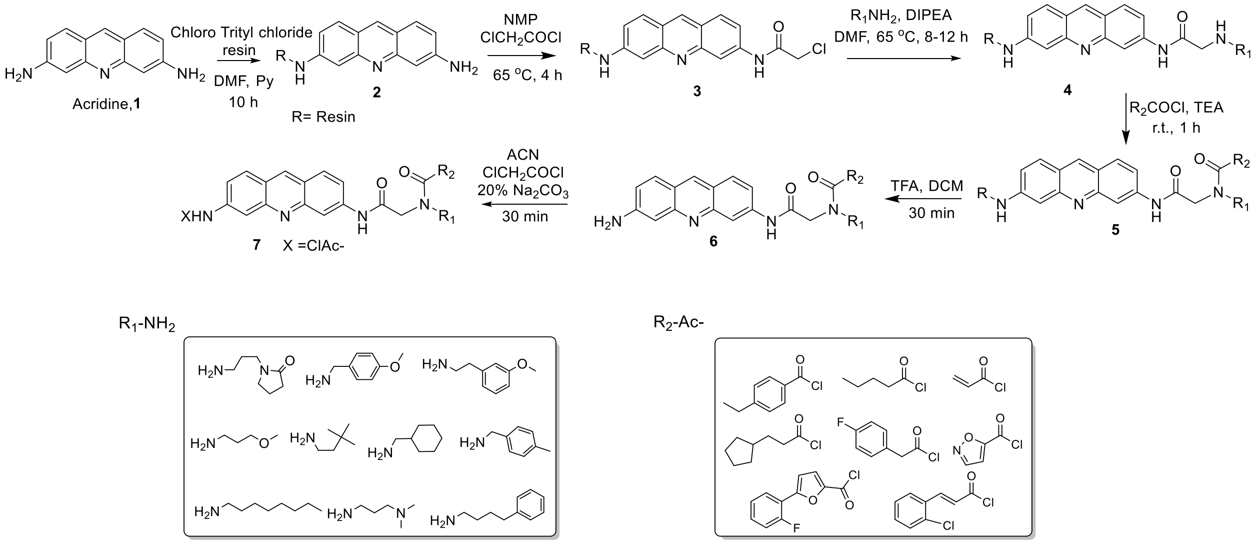

Supplementary Figure 3. Synthetic scheme of the Acridine library.

Supplementary Figure 4.

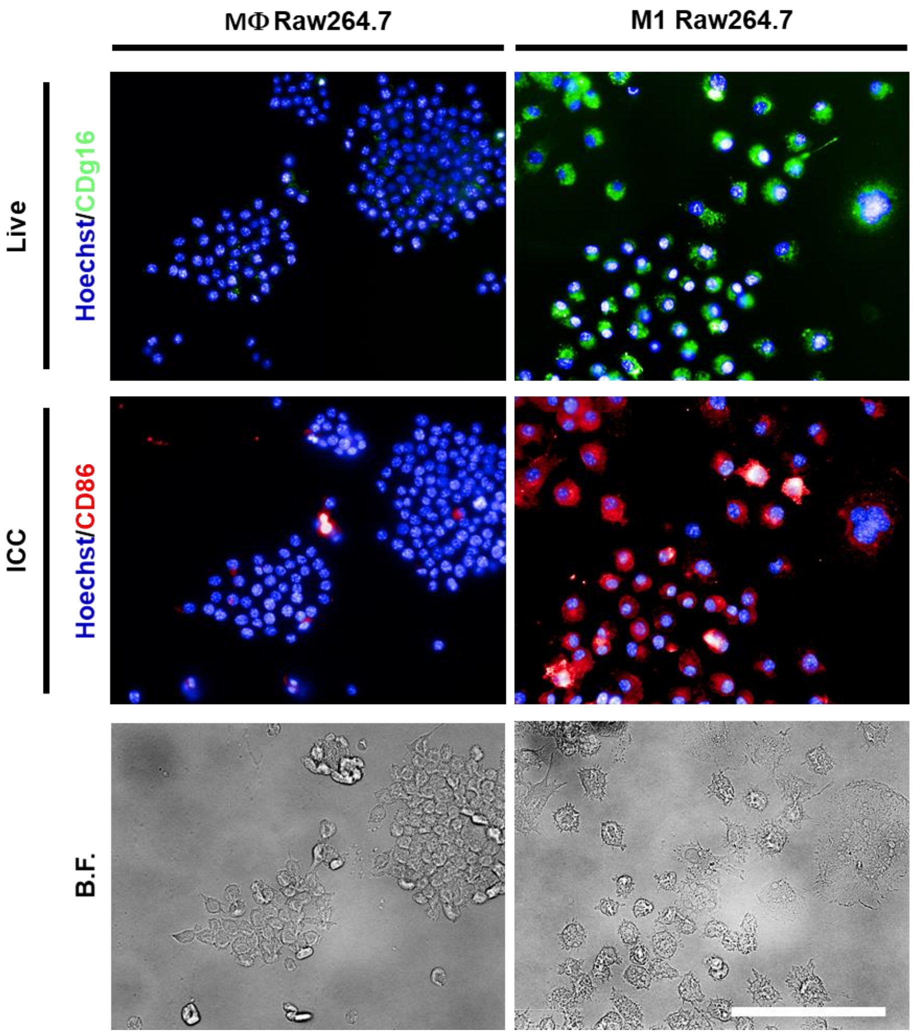

Supplementary Figure 4. CDg16 labels CD86 positive M1 macrophages. Fluorescence images of live non-activated (Mϕ) and M1 Raw264.7 cells after incubating with CDg16 (1 μM) and Hoechst33342 (1 μg/mL) for 1 hour. CD86 immunocytochemistry (ICC) images was taken after performing a general ICC protocol. B.F., bright fileld; Scale bar, 200 μm.

Supplementary Figure 5.

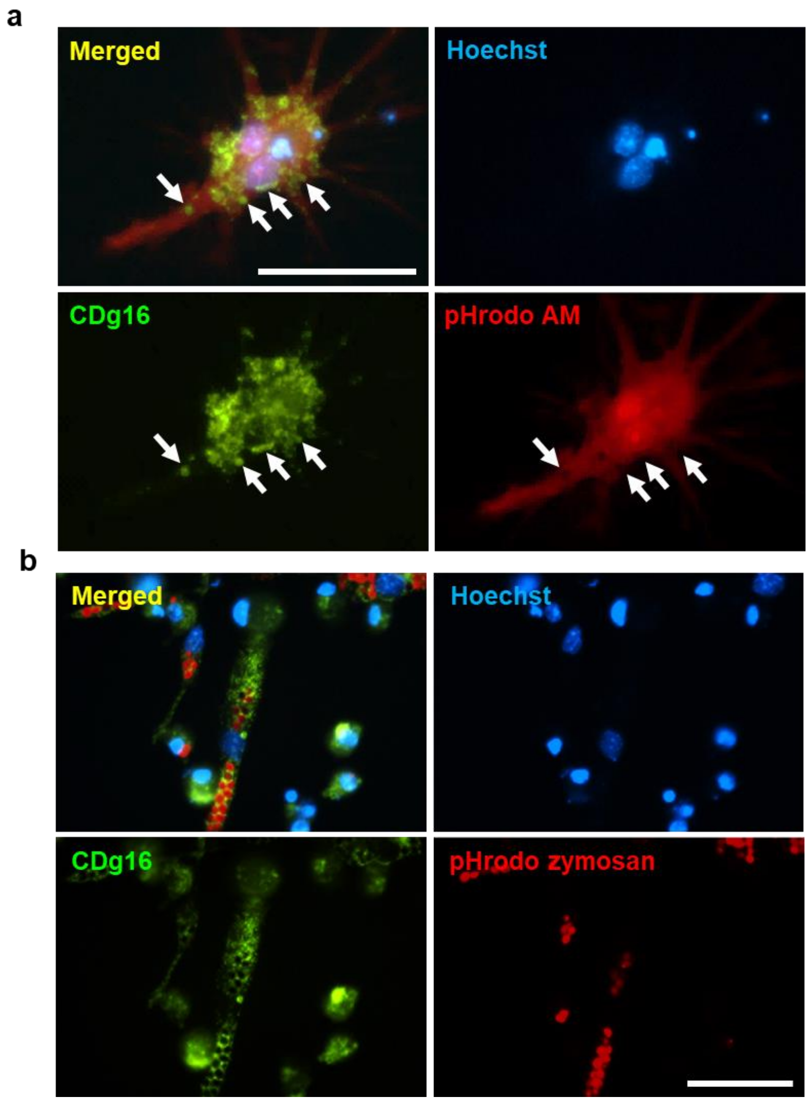

Supplementary Figure 5. The pH independent lysosomal staining of CDg16. a, M1 polarized Raw264.7 cells were incubated with pHrodo^{TM} Red AM for 20 minutes to detect the cytoplasmic area of low pH, followed by CDg16 staining (1 µM). White arrows, pHrodo Red AM-unstained CDg16^{bright} vesicles. **b,** M1 macrophages were incubated with pHrodo^{TM} Red conjugated Zymosan Bioparticles^{TM} to detect low pH phagocytotic vesicles for 1 hour, followed by CDg16 staining. Scale bar, 50 µm.

Supplementary Figure 6.

a

S/N	Compound	ClogD (at pH 7.4)	ClogD (at pH 4.5)	tPSA
1	Acridine Orange	2.93	2.01	19.4
2	CDg16	3.31	1.78	114.4

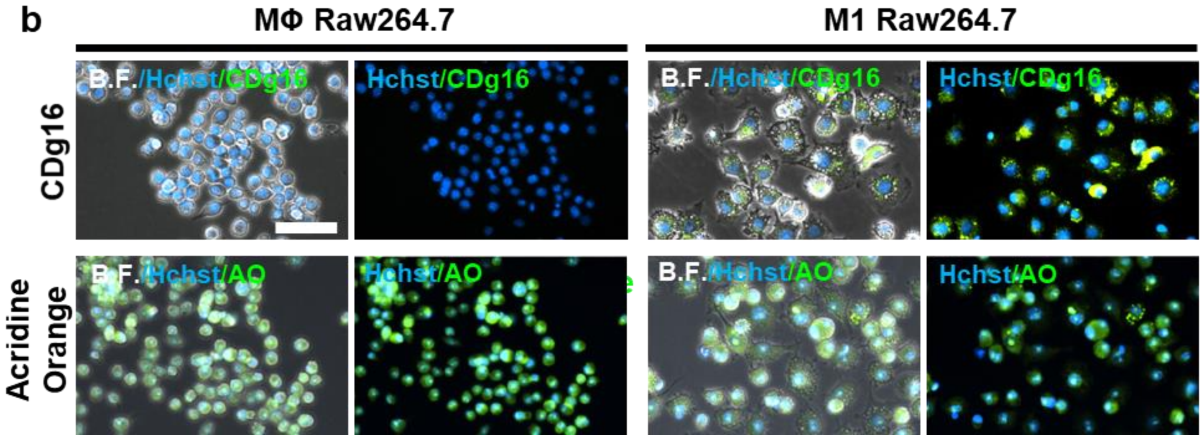

Supplementary Figure 6. The staining pattern of CDg16 and acridine orange (AO) in control and M1 macrophages. a, CLogD (at pH7.0) and tPSA values of acridine orange and CDg16 were calculated by Chemicalize program from ChemAxon. **b,** The staining pattern of CDg16 (1 μM) and acridine orange (AO, 500 nM) were compared between non-activated (Mϕ Raw264.7) and M1 macrophages (M1 Raw264.7). Note that the staining of AO labels both Mϕ and M1 Raw264.7 cells unlike to the specific staining of CDg16 to M1. Hchst, Hoechst; AO, acridine orange. Scale bar, 50 μm.

Supplementary Figure 7.

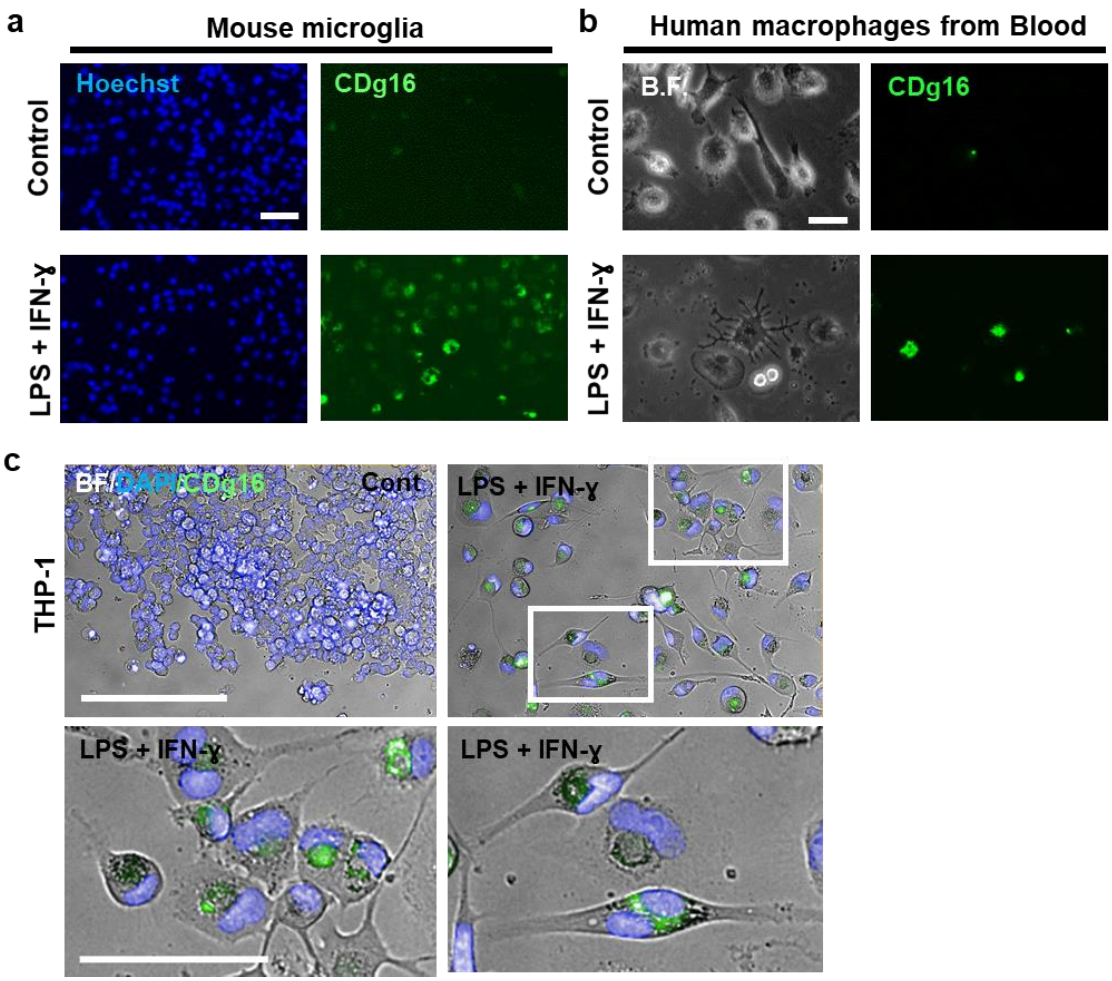

Supplementary Figure 7. CDg16 stain pattern in mouse microglia, human macrophages and THP-1 cell line. Mouse microglia (**a**), human macrophages (**b**) and THP-1 (**c**) cell line were activated by LPS and IFNγ and examined by live cell imaging after incubation with CDg16. Note, M1 (LPS + IFNγ) showed high CDg16 fluorescence signal. Scale bars, 100 μm (a,b,c-lower) and 200 μm (c-upper).

Supplementary Figure 8.

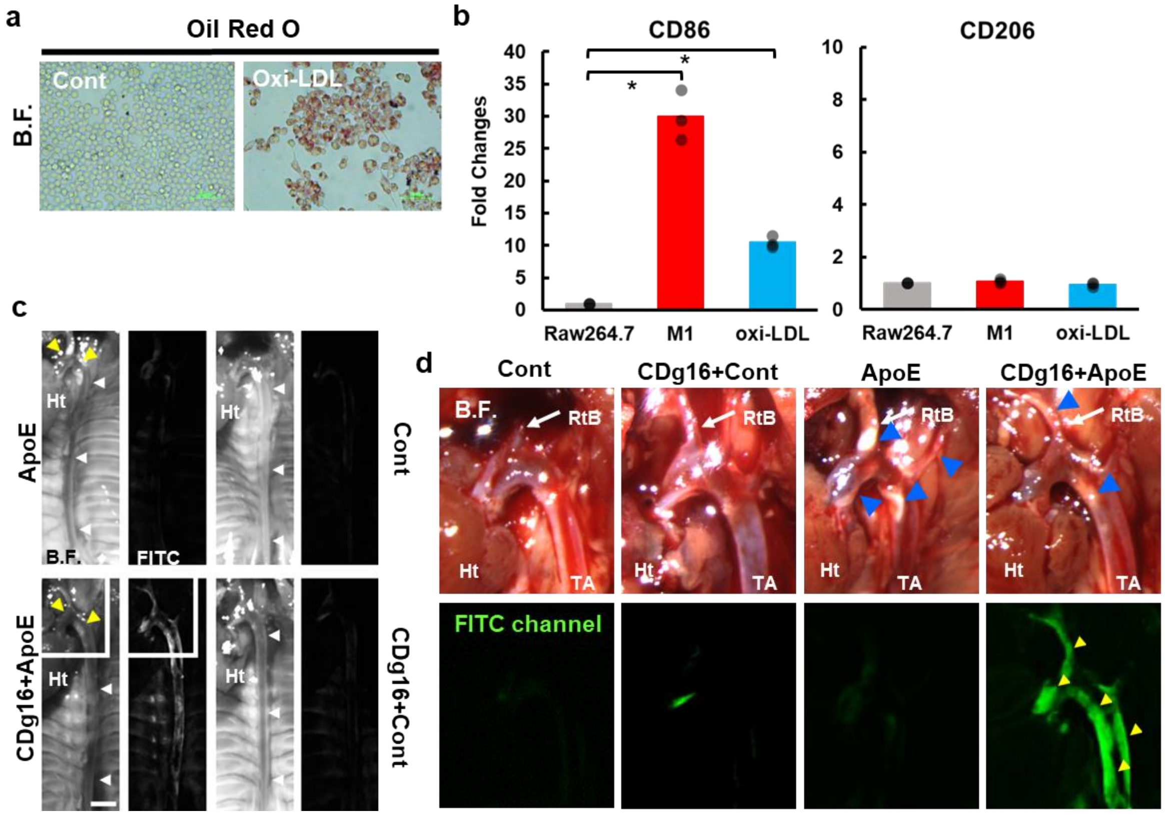

Supplementary Figure 8. Application of CDg16 for visualizing atherosclerosis. a, Oil Red O staining in control (Cont) and the oxi-LDL treated Raw264.7 cells (oxi-LDL). **b,** CD86 and CD206 mRNA expression in oxi-LDL-treated Raw264.7 cells (120 µg/mL). RT-PCR analysis (*, $p < 0.01$). Average values with each data-points (dots) (N=3). **c,** CDg16 signals in the thoracic region of non-injected Cont, CDg16-injected control (CDg16+Cont), non-injected ApoE KO (ApoE) and CDg16-injected ApoE KO (CDg16+ApoE) mice. Fluorescent stereomiroscopy. Yellow arrowheads, plaques in aorta arch; White arrowheads, thoracic aorta. **d,** Comparison of CDg16 signals of Cont, CDg16+Cont, ApoE, and CDg16+ApoE aorta arches. Yellow arrowheads, CDg16 staining plaques under FITC channel; Blue arrowheads, plaques under bright field. B.F., bright field; Ht, heart; RtB, right brachiocephalic artery; TA, thoracic artery.

Supplementary Figure 9.

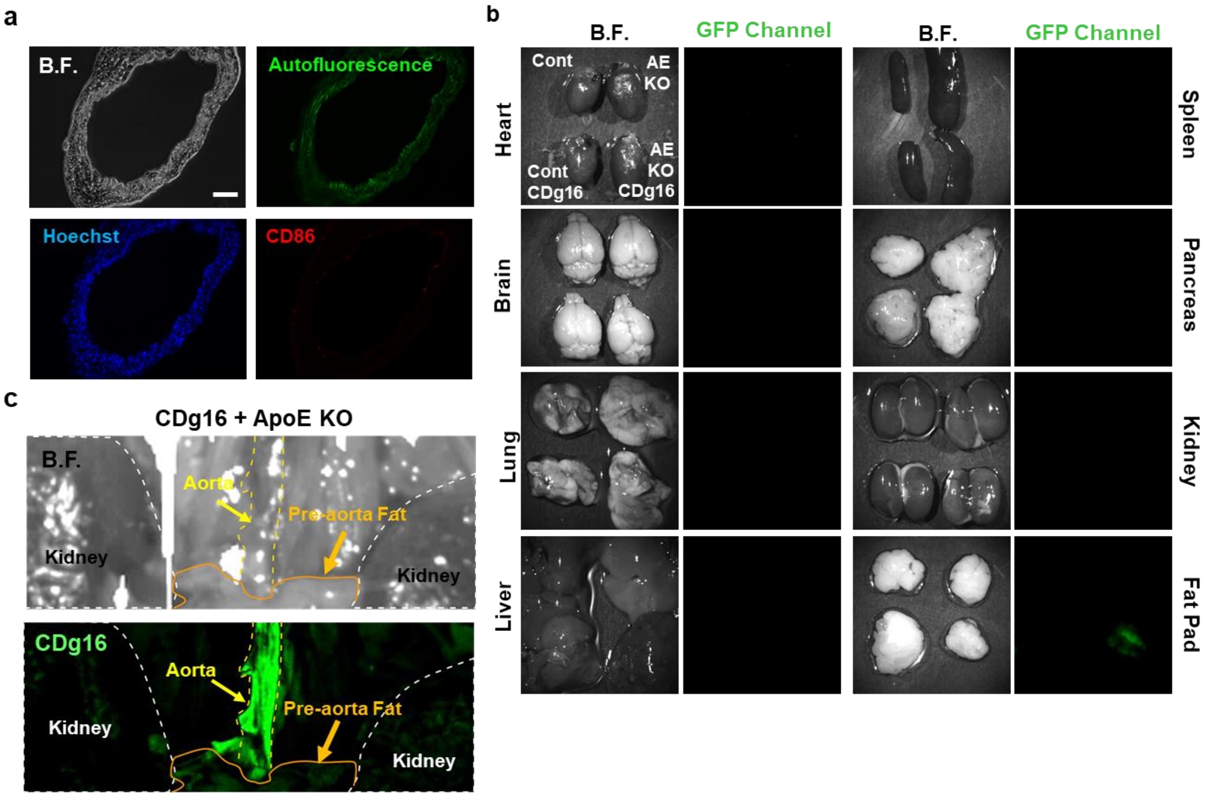

Supplementary Figure 9. CDg16 bio-distribution in atherosclerosis disease model. **a,** Fluorescent level of control blood vessel under FITC channel (autofluorescence) and Cy5 channel after CD86 IHC. **b,** CDg16 staining pattern in other organs of Cont, CDg16+Cont, ApoE (AE KO) and CDg16+ApoE (AE KO CDg16) mice. CDg16 signal was examined by the fluorescent stereomicroscopy under GFP channel. Faint CDg16 signals were observed in fat pad. **c,** The relative CDg16 intensity between aorta (green) and pre-aorta fat (orange color boundary). Note, CDg16 staining aorta showed much stronger CDg16 signals than pre-aorta fat.

Supplementary Figure 10.

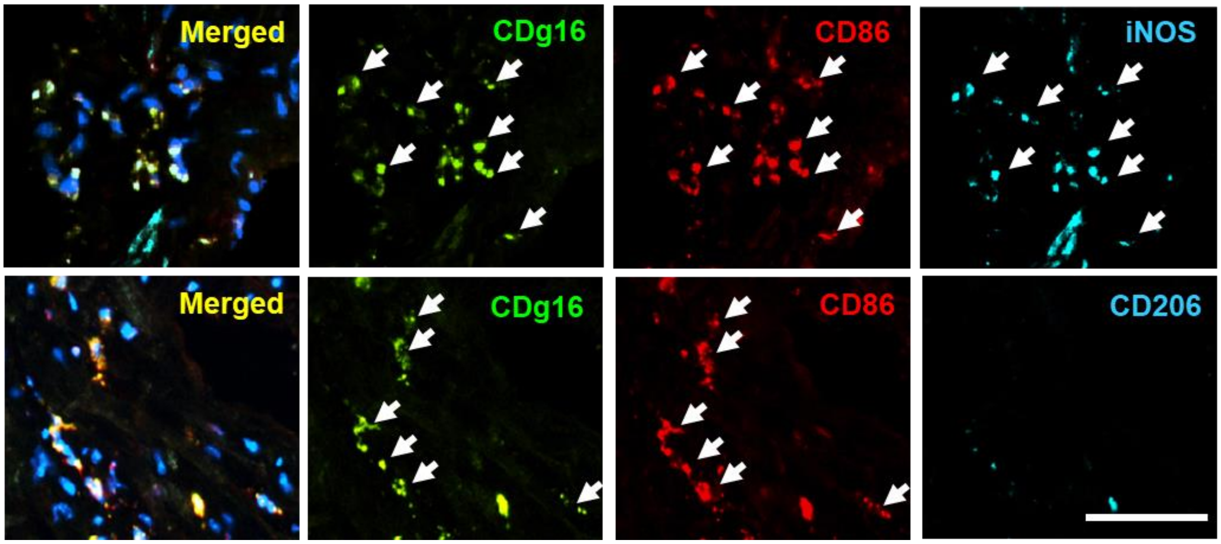

Supplementary Figure 10. Histological evaluation of CDg16 positive cells in atherosclerotic plaque. Cryosectioned CDg16-injected aorta from the atherosclerosis-bearing mouse (ApoE-/-) was immunolabeled with antibodies to CD86, iNOS (M1 marker) and CD206 (M2 marker). White arrows in the upper panel are the triple-positive M1 cells with CDg16, CD86 and iNOS in the plaque area of aorta. CD206 positive cells were rarely stained in plaque (lower panel). Scale bar, 50 µm.

Supplementary Figure 11.

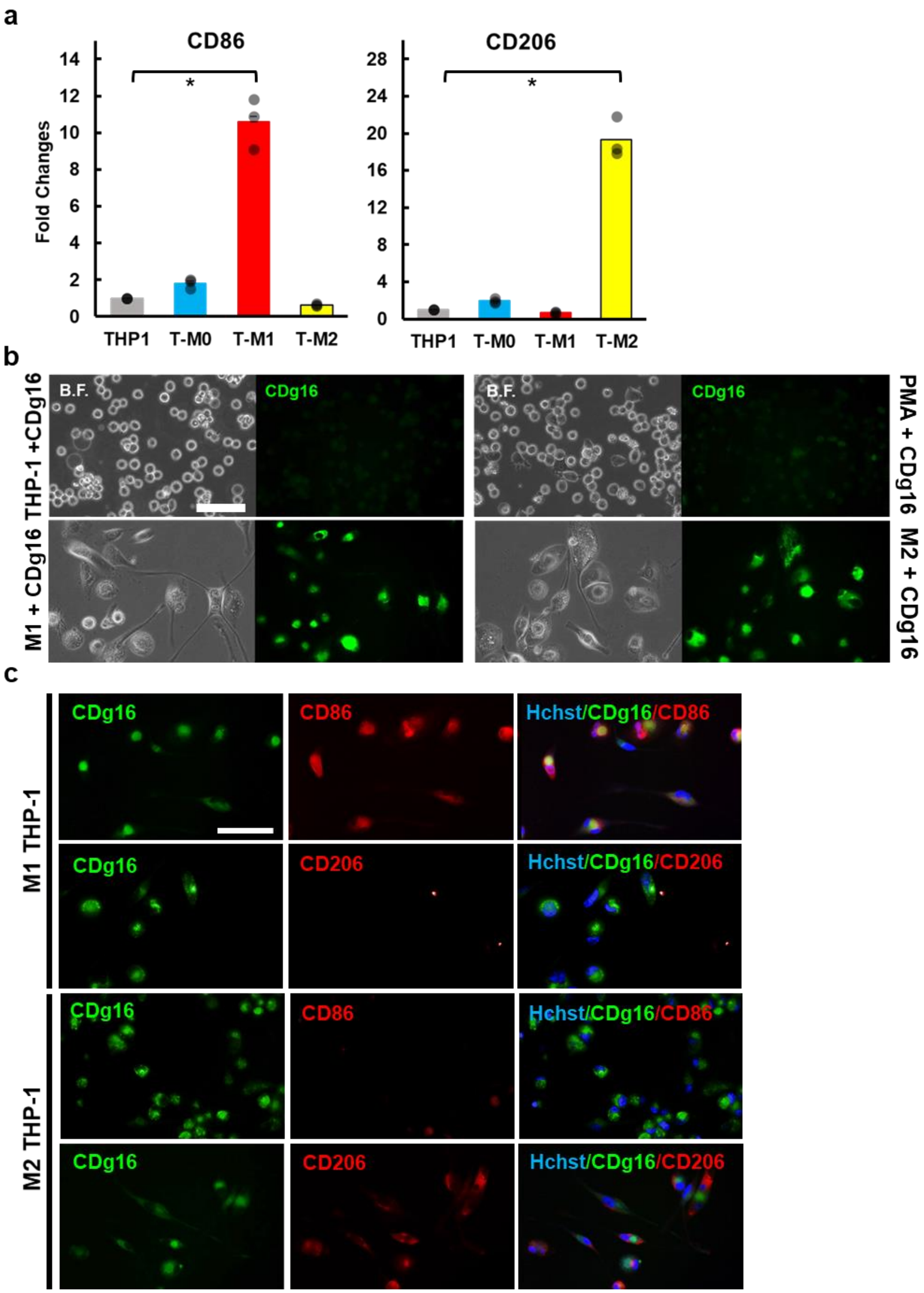

Supplementary Figure 11. CDg16 staining in M1 and M2 activated THP-1 cells. a, Gene expression of CD86 and CD206 in the M1 and M2 activated THP-1 cells. M0, M1, and M2 differentiation was induced by incubation with PMA (phorbol 12-myristate 13-acetate, 50 nM), LPS (100 ng/mL) and IFNγ (20 ng/mL), and IL-4 (20 ng/mL) and IL-13 (20 ng/mL) to THP-1 cells, respectively. RT-PCR analysis. **b,** CDg16 stains both M1 and M2 activated THP-1 cells. CDg16, 500nM. **c,** Evaluation of M1 and M2 activation of THP-1 cells by CD86 and CD206 immunocytochemistry. T-M0, M0 THP-1; T-M1, M1 activated THP-1; T-M2, M2 activated THP-1; Hchst, Hoechst. Scale bars, 50 μm.

Supplementary Figure 12.

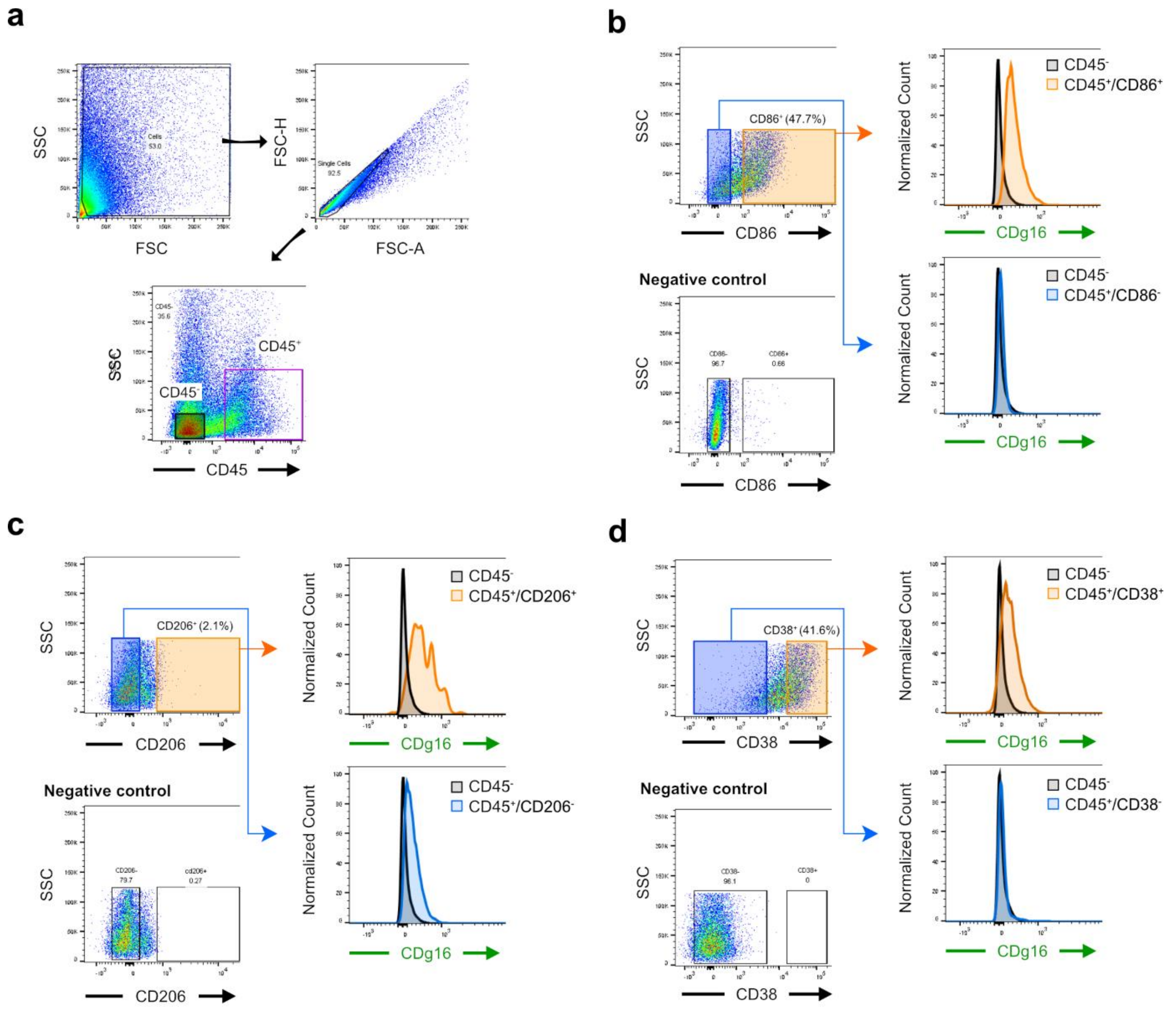

Supplementary Figure 12. Quantitative analysis of CDg16-stained cells in the aorta of ApoE$^{-/-}$. **a,** Singlet gating strategy of the dissociated aorta cells. **b,** CDg16 fluorescence intensity was analyzed in the CD45^{+}/CD86^{+} M1 macrophages (Orange) and the CD45^{+}/CD86^{-} leukocytes. **c,** Analysis of the CD45^{+}/CD206^{+} M2 macrophages (Orange) and the CD45^{+}/CD206^{-} leukocytes. **d,** Analysis of the CD45^{+}/CD38^{+} M1 macrophages (Orange) and the CD45^{+}/CD38^{-} leukocytes. **b-d,** The other CD45^{-} cells (Black) were used as negative control for the CD45^{+} groups.

Supplementary Figure 13.

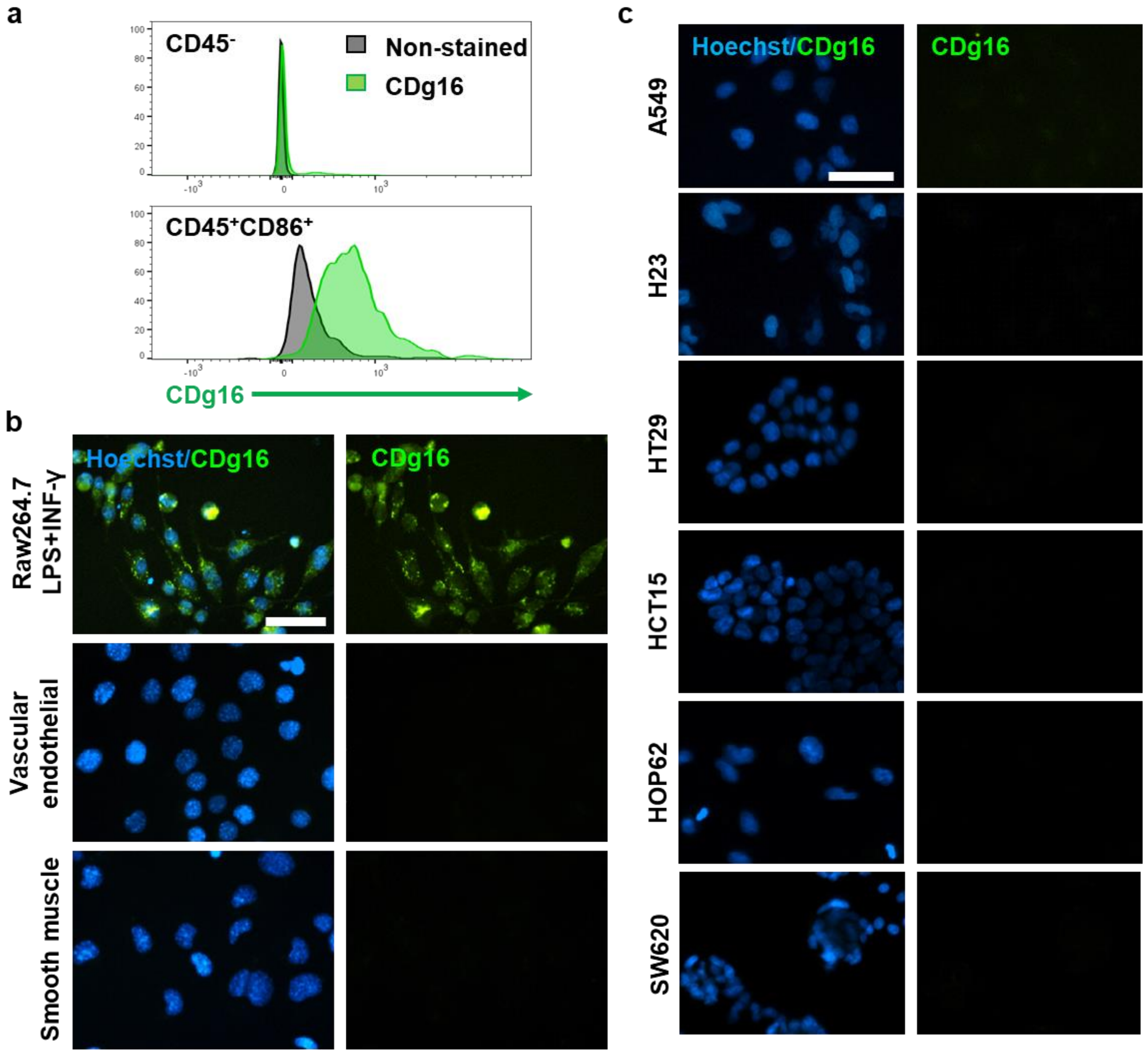

Supplementary Figure 13. CDg16 specificity to activated macrophages compared to other cell types of aorta. a, CDg16 stain M1 macrophages over the autofluorescent signals of activated macrophages. $CD45^-$, non-leukocytes in aorta; $CD45^+/CD86^+$, M1 macrophages in aorta. **b,** No staining of CDg16 in mouse vascular endothelial cells (2H-11) and smooth muscle cells (MOVAS) compared to M1 activated Raw264.7 cells. **c,** No staining of CDg16 in the epithelial cell derived lung cancer (A549, H23, HOP62) and the epithelial cell derived colon cancer (HT29, HCT15, SW620) cell lines. Scale bars, 50 µm.

Supplementary Figure 14.

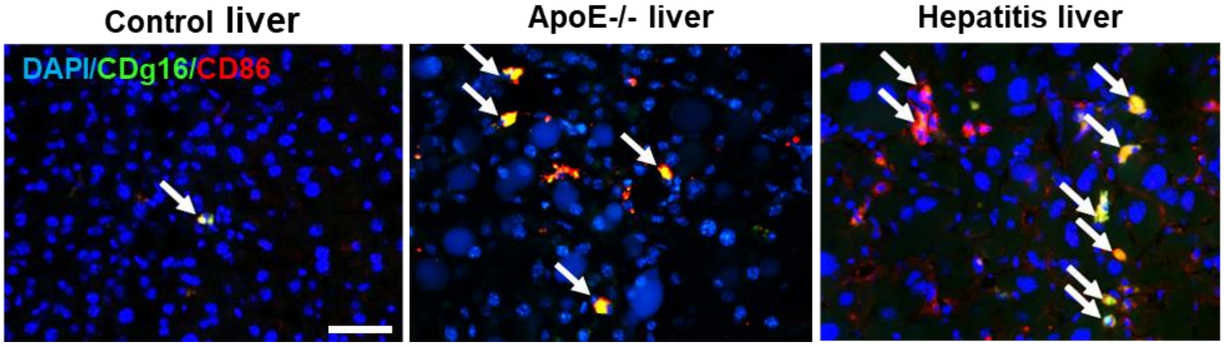

Supplementary Figure 14. Visualizing inflamed liver using CDg16. CDg16 were injected intravenously to control, ApoE-/- and hepatitis mouse at 1 hour before the liver enucleation. Cryosectioned livers were immunostained with CD86 antibody. White arrows, CDg16 and CD86 double-positive cells in liver; Blue, nuclei; Scale bar, 100 µm.

Supplementary Figure 15.

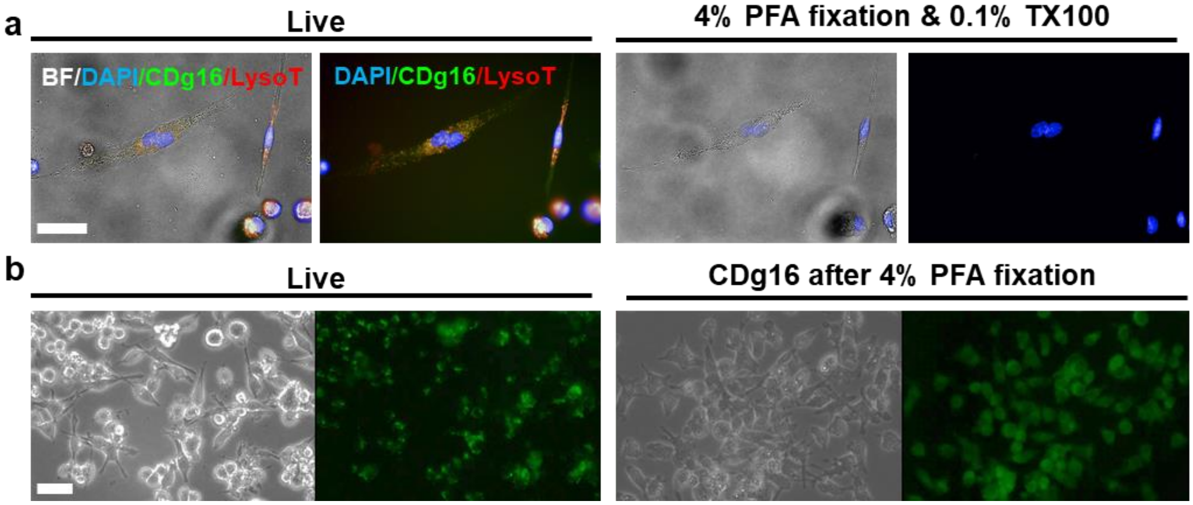

Supplementary Figure 15. Differential staining pattern of CDg16 in live and dead M1 macrophages. **a,** Double labeling of live M1 macrophages with CDg16 and LysoTracker (LsyoT) (Live) and its disappearance after 4% PFA fixation and 0.1% Triton X-100 permeabilization (dead cells, 4% PFA fixation & 0.1% TX100). **b,** Different subcellular labeling was observed depending on the cell status at the CDg16 treatment. Vesicular and cytosolic staining pattern was observed when CDg16 incubated to live and 4% PFA fixed M1 macrophages (CDg16 after 4% PFA fixation), respectively. Scale bar, 100 µm.

Supplementary Figure 16.

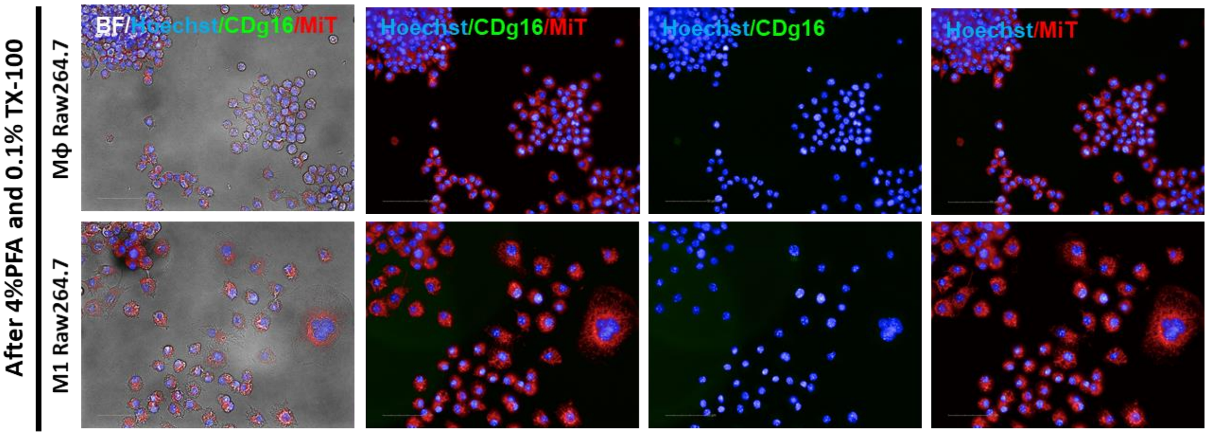

Supplementary Figure 16. Reversible staining of CDg16 by fixation and permeabilization. Hoechst33342, CDg16 and MitoTracker was co-treated to live Mϕ (upper panel) and M1 Raw264.7 (lower panel). The stained cells were fixed and permeabilized with 4% PFA and 0.1% TritonX-100. Note that the CDg16 fluorescent signal (Green) in M1 cells were disappeared, but the Hoehcst33342 (Blue) and MiT (Red) signals still remained in both Mϕ and M1 cells. BF, bright field; Hoechst, Hoechst33342; MiT, MitoTracker; Scale bar, 50 µm.

Supplementary Figure 17.

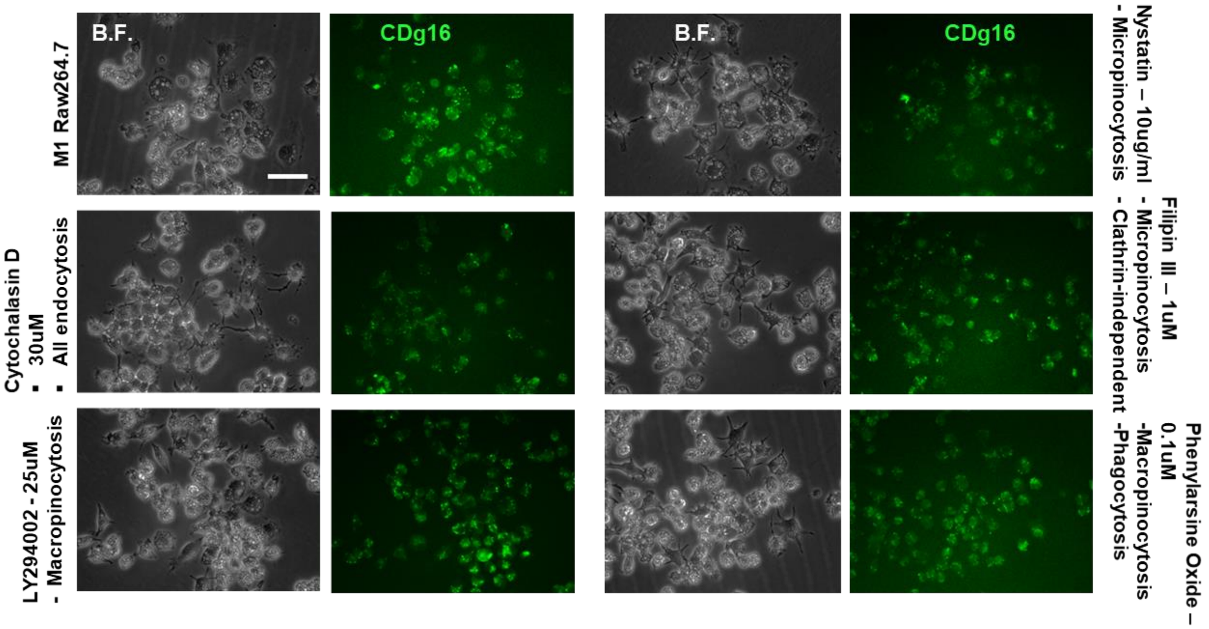

Supplementary Figure 17. Inhibition of endocytosis has no effect on the staining of CDg16. The indicated endocytosis inhibitors were pre-treated to activated macrophages (Mϕ*) and the CDg16 stain patterns were examined. The vesicular staining pattern of CDg16 in live Mϕ* cells was not affected by any of the endocytosis inhibitors. Scale bar, 100 µm.

Supplementary Figure 18.

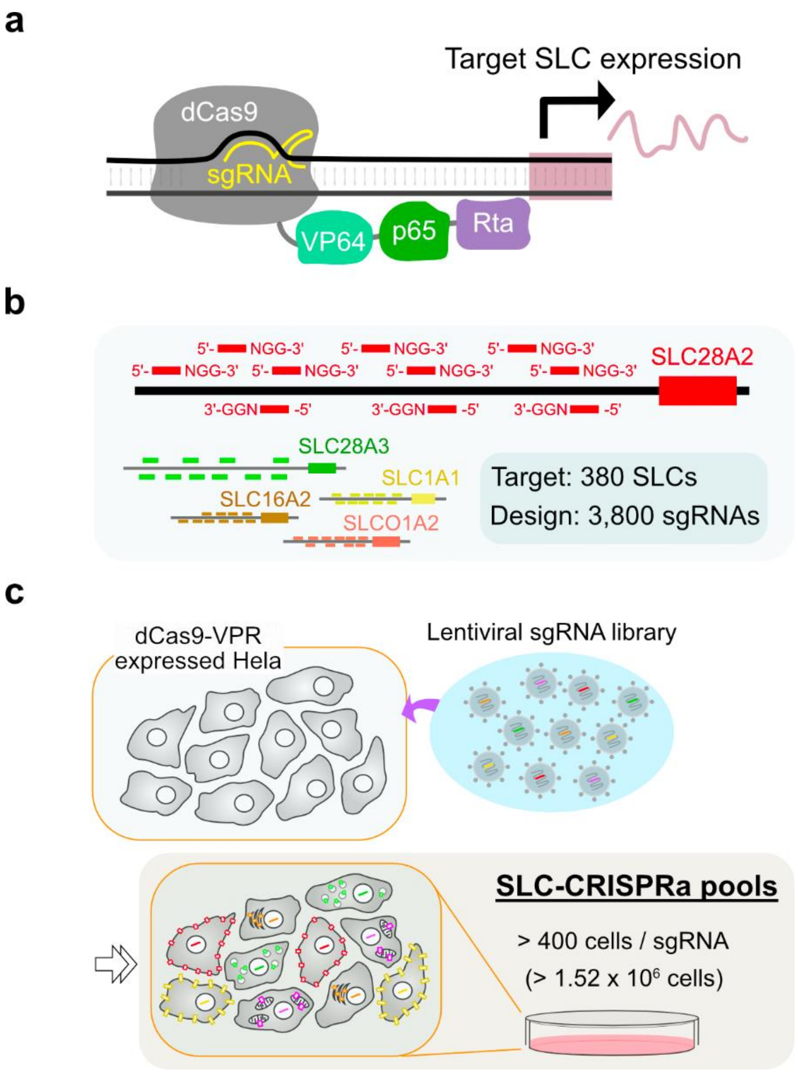

Supplementary Figure 18. Construction of the SLC-CRISPRa screening system. a, Schematic figure of an SLC gene activation by co-expression of dCas9-VPR and a sgRNA. Pink box, transcription initiation site. **b,** Schematic summary of the sgRNA library targeted to the promoters of 380 SLC genes. **c,** Scheme for generation of the SLC-CRISPRa pools using HeLa cells.

Supplementary Figure 19.

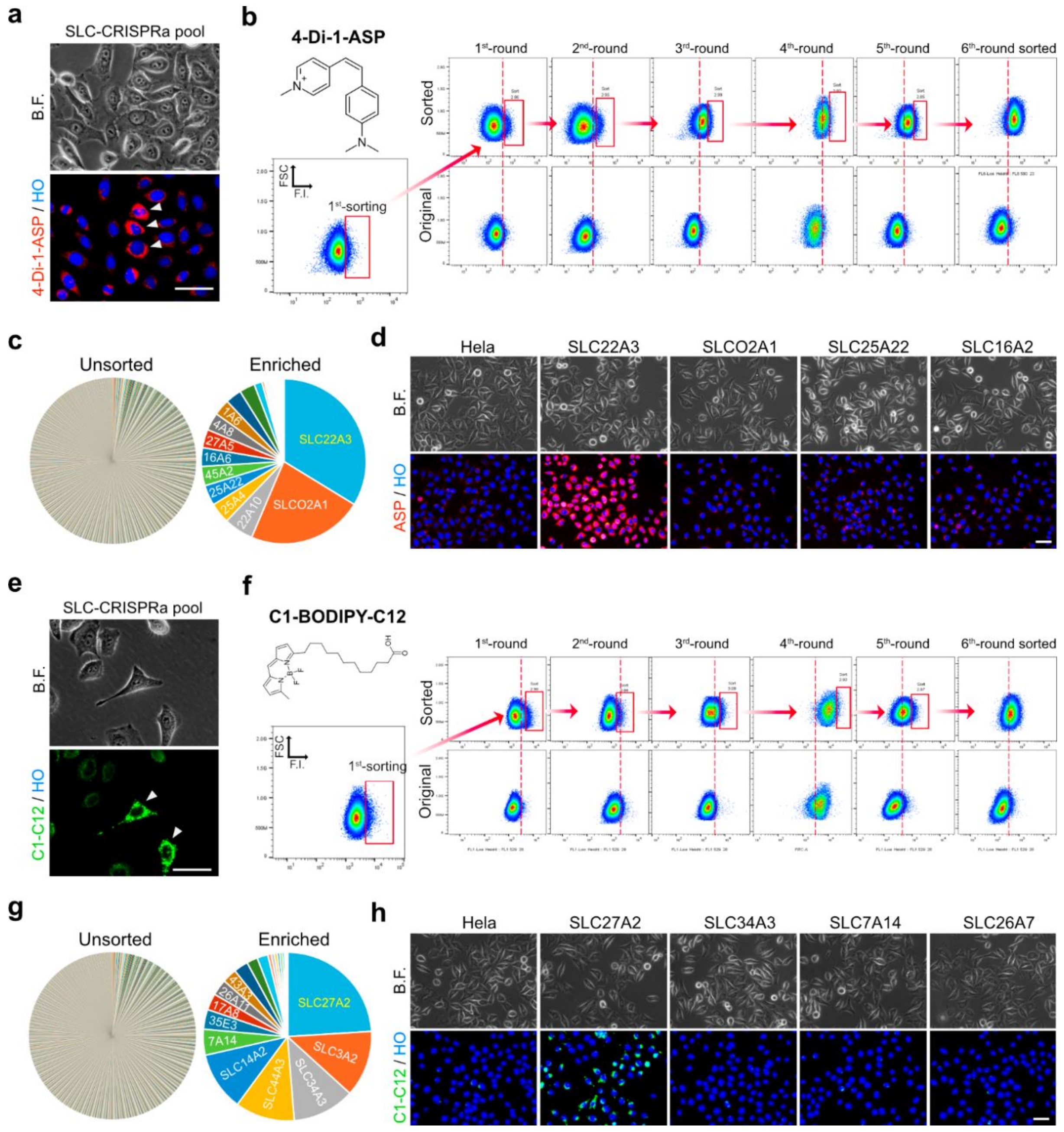

Supplementary Figure 19. Evaluation of the SLC-CRISPRa screening system. The evaluation was conducted with two fluorescence probes, 4-Di-1-ASP (a-d) and C1-BODIPY-C12 (e-h). **a and e,** The SLC-CRISPRa Hela pools were stained with 4-Di-1-ASP (a) or C1-BODIPY-C12 (e). **b and f,** The chemical structure of probes and the fluorescence densitometry of the enriched populations while 6th-round enrichment processes for selection of the 4-DI-1-ASP (b) or C1-BODIPY-C12 (f) brightly-stained population (red box, expanded top 3%-stained population). FSC, forward scattering; F.I., fluorescence intensity; Original, original SLC-CRISPRa pool. **c and g,** Proportions of the top 1,000 highly enriched sgRNAs in the unsorted and the 6-round enriched population (Enriched) of 4-Di-1-ASP (c) or C1-BODIPY-C12 (g). The NGS counts of each shRNAs targeted to a particular SLC gene was presented as percentages in a pie chart. **d and h**, Fluorescence imaging of wild-type and the individual SLC activated Hela cells by co-expression of the single sgRNA and dCas9-VPR. Fluorescence staining was performed by one-hour incubation of a probe (1 µM). Hoechst33342 (HO) (1 µg/mL) was co-treated with a probe to label nuclei of live cells. ASP, 4-Di-1-ASP; C1-C12, C1-BODIPY-C12; B.F., bright-field images; Scale bars, 20 µm (a, e) or 50 µm (d and h).

Supplementary Figure 20.

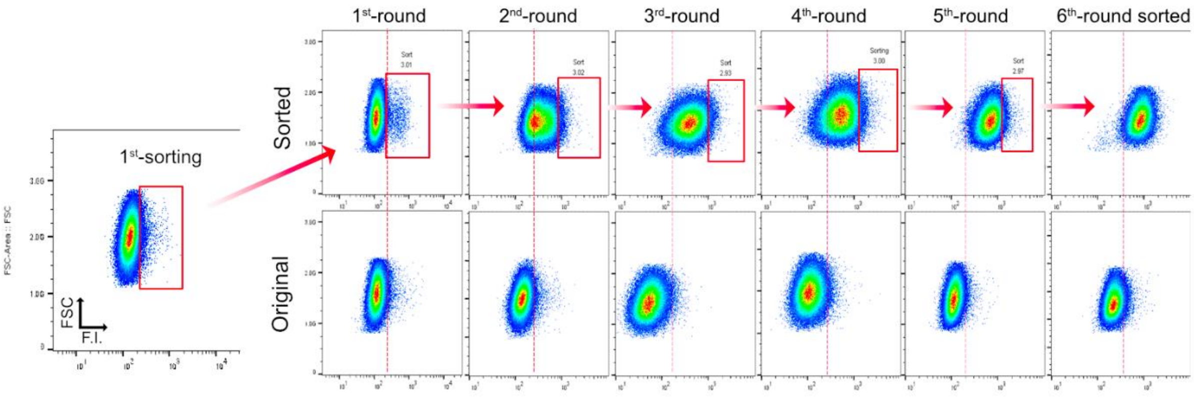

Supplementary Figure 20. Enrichment of the CDg16 brightly stained SLC-CRISPRa subpopulation. Fluorescence densitometry during the 6th-round enrichment process of CDg16. CDg16 was treated for one hour with 200 nM concentration. Sorted, expanded populations of the top 3% of brightly stained mother population (red box); Original, SLC-CRISPRa pool; FSC, forward scatter; F.I., CDg16 fluorescence intensity.

Supplementary Figure 21.

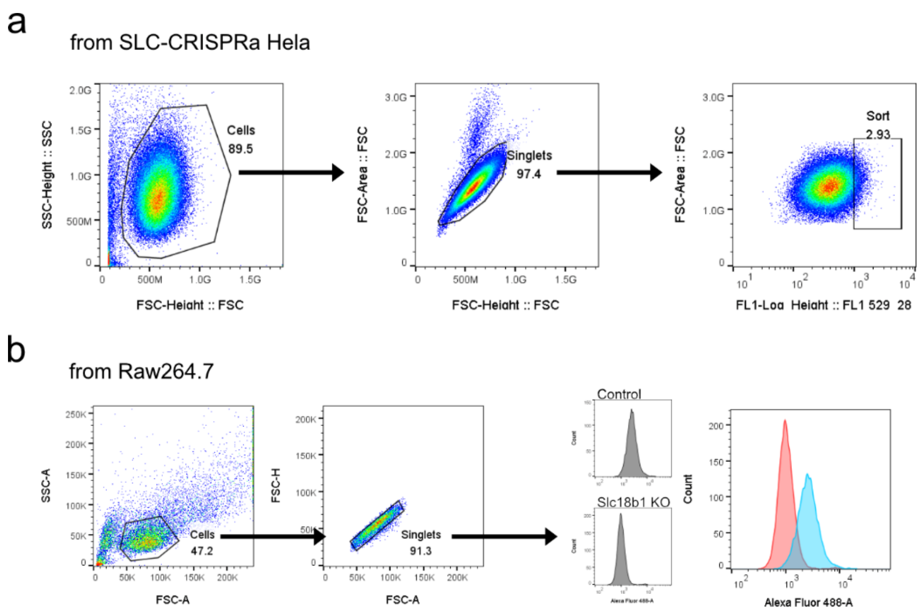

Supplementary Figure 21. Flow cytometry and FACS gating strategy. a, FACS gating strategy for SLC-CRISPRa Hela. The live cell populations selected with the indicated live FSC-H/SSC-H gating (Cells, 83–90% of the total events) were sub-gated with FSC-A/FSC-H to discriminate single cell population from aggregates (Singlets, 95-98% of the parental populations). The singlets were used for FACS based on the CDg16 signal intensity (the highest 3% of the CDg16-stained singlets). The indicated sample is 3rd-round sorted populations stained with CDg16. **b,** Flow cytometry gating strategy for Raw264.7 cells. The live cells selected with FSC-A/SSC-A gating (Cells, 25-50%) were sub-gated with FSC-A/FSC-H for singlets (89-92%). The CDg16 intensity of singlets was analyzed with Alexa Fluor 488-A channel. Pseudocolor plots were used to display the gating.

Supplementary Figure 22.

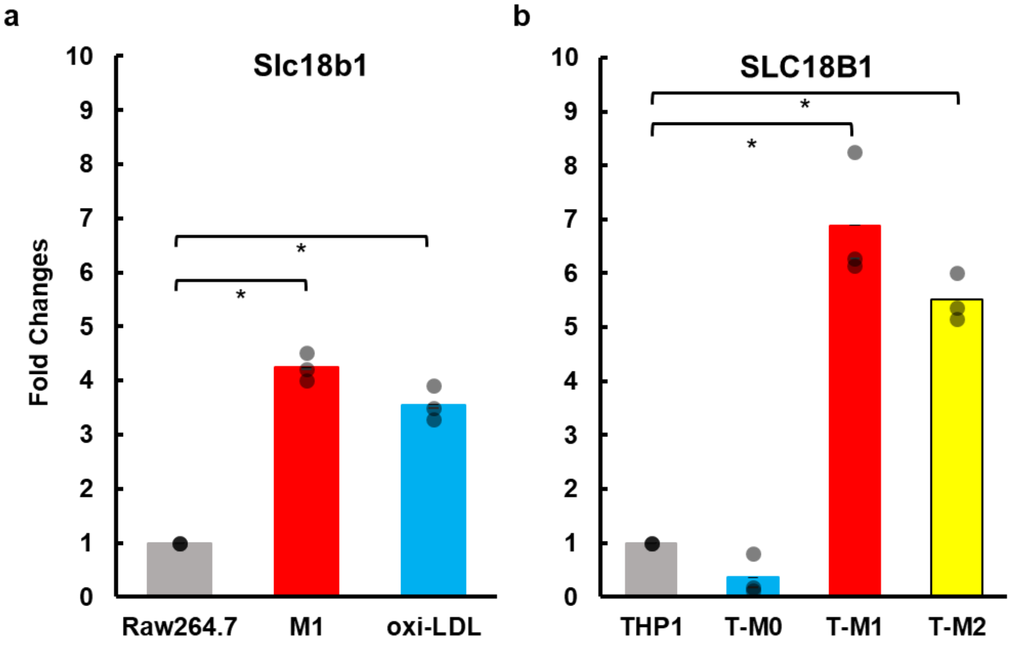

Supplementary Figure 22. The gene expression patterns of Slc18b1/SLC18B1 in various activation states of Raw264.7 and THP-1 cells. The gene expression levels of Slc18b1 (a) and SLC18B1 (b) were examined in control, M1, and oxi-LDL-treated mouse Raw264.7 cells (a) and control, M1, and M2 human THP-1 cells (b) by RT-PCR. *, $p < 0.01$; M1, M1 Raw264.7; oxi-LDL, oxi-LDL-treated Raw264.7; T-M0, PMA-treated THP-1; T-M1, M1 THP-1; T-M2, M2 THP-1.

Supplementary Methods

Cell culture

To test CDg16 behavior in the different cell lines, human monocyte cell line (THP-1, ATCC® TIB-202™, human monocyte), mouse vascular endothelial cell line (2H-11, ATCC® CRL-2163), mouse smooth muscle cell line (MOVAS, ATCC® CRL-2797), epithelial cell derived lung cancer cell lines (A549, H23, HOP62) and epithelial cell derived colon cancer cell lines (HT29, HCT15, SW620) were cultured with 90% Dulbecco's modified Eagle's medium with 4.5 g/L glucose and 10% heat-activated fetal bovine serum for CDg16 (1 μM) stain pattern.

Immunofluorescence staining

Cultured cells, primary cells from aortas and cryo-tissue sections were fixed in 4% paraformaldehyde for 15mintes. The samples were incubated with 1% bovine serum albumin for blocking non-specific binding. For mouse M1 or M2 activated macrophages, AlexaFluor® 594 conjugated anti-B7-2/CD86 antibody (dilution 1:100, R&D Systems®, FAB741T), AlexaFluor® 647 conjugated anti-iNOS antibody (dilution 1:100, Abcam®, ab209027), APC conjugated anti-CD206 (MMR) antibody (dilution 1:50, BioLegend®, 141708), PE/Cy7 conjugated anti-CD38 (dilution 1:1000, BioLegend®, 102717) and PerCP/Cy5.5 conjugated anti-CD45 antibody (dilution 1:1000, BioLegend®, 147706) were incubated for 30min at 37°C or overnight at 4°C for staining activated macrophages. For human M1 or M2 activated macrophages, AlexaFluor® 594 conjugated anti-human CD86 (dilution 1:100, R&D Systems®, FAB141T) and AlexaFluor® 594 conjugated anti-

human CD206 (dilution 1:100, BioLegend®, 321116) were incubated overnight at 4°C for staining human activated macrophages. The stained cells were observed by The BD LSRFortessa™ or Eclipse Ti-E Microscopy (Nikon).

CDg16 stain pattern in low pH phagocytotic vesicles

pHrodo™ Red Zymosan Bioparticles™ (pHrodo-Zymosan, ThermoFisher Scientific) can detect low pH phagocytotic vesicles because of turning on pHrodo under low pH and zymosan bacterial particles for phagocytotic vesicles. As we followed the protocol of the company, activated Raw264.7 cells by LPS and IFN-γ in the 12 well plate were incubated in Opti-MEM® media for 30min before pHrodo-Zymosan treatment. 100 µL of pHrodo-Zymosan was added into each well for 1hr. Then, CDg16 (1 µM) was added into each well and incubated for 1hr. All incubation was done in a humidified incubator with 5% CO2 at 37°C. Each well was washed with Opti-MEM® media, three times, and observed by Eclipse Ti-E Microscopy (Nikon).

Measurement of nitric oxide

The amount of nitric oxide formation was calculated by measuring optical density of samples at 540 nm after adding the cell culture medium (50 µL) with an equal volume of Griess reagent (0.1% naphthylethylene diamine, 1% sulfanilamide, 2.5% H3PO4).

Flow cytometry for atherosclerotic aorta cells

To analyze the cells of in vivo aorta, mice were euthanized and circulating blood was removed through cardiac puncture. Aorta was carefully removed from the mice under a

stereomicroscope to minimize contamination of surrounding connective tissue. The removed aorta was washed with HBSS buffer (contained 1% BSA, 5% glucose, 20 M adenosine) and dissected well with scissors. The chopped tissues were further digested with collagenase type I (Worthington Biochemicals, 2.5 mg/mL) and DNase I (Worthington Biochemicals, 60 U/mL) for 30 minutes at 37°C. Dissociated single cells were collected by using 40 µm cell strainer. Single cells of aorta were resuspended to 2 mL of the HBSS buffer after centrifugation (400xg for 5 minutes) and proceeded for immunolabeling.

M1 and M2 macrophages were labeled by a set of antibodies as followed: CD45-PerCP-Cy5.5 (Biolegend, 147706, 1:1,000), CD86-Alexa Fluor 594 (R&D systems, FAB741T, 1:100), CD38-PE-Cy7 (Biolegend, 102717, 1:1,000), CD206-APC (Biolegend, 141708, 1:500). After incubating dissociated aorta cells with the antibodies for 1 hour, CDg16 (500 nM) were added to the cells for 30 minutes before analysis. Flow cytometry was performed by using LSRII flow cytometer.

AD and ADCA small fluorescent chemical library

All reactions were performed in oven-dried glassware under a positive pressure of nitrogen. Unless otherwise noted, starting materials and solvents were purchased from Aldrich and Acros organics and used without further purification. Analytical TLC was carried out on Merck 60 F254 silica gel plate (0.25 mm layer thickness) and visualization was done with UV light. Column chromatography was performed on Merck 60 silica gel (230-400 mesh). NMR spectra were recorded on a Bruker Avance 500 NMR spectrometer. Chemical shifts are reported as *δ* in units of parts per million (ppm) and coupling constants are reported as a *J* value in Hertz (Hz). Mass of all the compounds was determined by LC-MS of Agilent Technologies with an electrospray ionization source. Spectroscopic measurements were performed on a fluorometer and UV/VIS instrument, BioTek Synergy 4 and Gemini XS fluorescence plate reader. The slit width was 1 nm for both excitation and emission. Relative quantum efficiencies were obtained by comparing the areas under the corrected emission spectrum. The following equation was used to calculate quantum yield

(1) $\Phi_x = \Phi_{st}(I_x/I_{st})(A_{st}/A_x)(\eta_x^2/\eta_{st}^2)$

where Φ_{st} is the reported quantum yield of the standard, *I* is the integrated emission spectrum, *A* is the absorbance at the excitation wavelength, and η is the refractive index of the solvents used (η = 1 if same solvent). The *x* subscript denotes unknown and st denotes standard. Acridine yellow was used as standard compound (e=39400 QY=0.47).

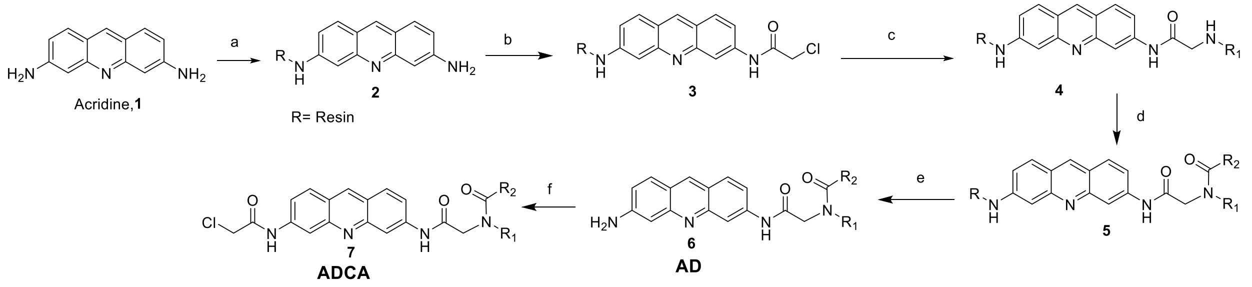

Supplementary Figure 23. Synthetic procedure of the AD and ADCA library. a. Chlorotrityl chloride resin (1g), DMF (30 mL), Pyridine (2.5 mL), r.t., 10 h; **b.** NMP (3.0 mL), $ClCH_2COCl$ (1.0 mL), 65 ^oC, 4 h; **c.** $R1NH_2$ (125 mg), DIPEA (1.0 mL), DMF, 65 ^oC, 8-12 h; **d.** R2COCl (125 mg), Pyridine (1.0 mL), r.t., 1 h; **e.** 2%TFA in DCM (5.0 mL), 30 min; f. Chloroacetyl chloride (2.5 equiv), acetonitrile (2.5 mL), saturated $NaHCO_3$ solution (3.0 mL), 0 ^oC, 30 min to 1h.

1. General synthetic procedure of AD library

<u>Synthesis of compound 2:</u> 2-Chlorotrityl chloride resin (1g, 1 mmole/g) was pre-swelled in 30 mL of DMF. To this resin suspension, diaminoacridine hydrochloride (550 mg) and Pyridine (2.5 mL) were added and fixed to a rotator mixture for spinning at room temperature. Spinning was stopped after 10 h and the reaction mixture was filtered using a 20 mL syringe having a frit, washed with DMF (50 ml), MeOH (50 ml) and DCM (50 ml), and dried under vacuum for 1hr. The dried resin was subjected to methanol capping procedure for deactivating the excess 2-Chlorotrityl chloride resin. DMF/MeOH mixture (80:20, 30 ml) was added to the resin mixture and was subjected to shaking at 250 rpm on a shaker for 1 h at room temperature. The resin was then filtered using a 20 mL syringe having a frit, washed with copious amount of solvents DMF (3 x 30 ml), MeOH (3 x 30 ml) and DCM (5 x 30 ml). The resin was then dried under high vacuum for 8 h and used for further reactions.

<u>Synthesis of compound 3:</u> To the resin 2 (300 mg) in 3.0 mL of *N*-Methyl pyrolidine (NMP), chloroacetyl chloride (1.0 mL) was added and the reaction mixture was subjected to shaking at 330-350 rpm at 65 ^oC.. After 4 h of constant shaking, the reaction mixture was cooled to room temperature, filtered using a 20 mL

syringe having a frit and washed with copious amount of solvents DMF (3 x 25 ml) and DCM (5 x 20 ml). The resin was dried under high vacuum for 8 h and used for further reactions.

Synthesis of compound 4: For the parallel synthesis of each member of library, the resin 3 (200 mg) was placed in a vial, 3 mL of DMF, amine (125 mg) were added followed by *N*, *N*-Diisopropylethylamine (1.0 mL). After shaking the reaction mixture at 330-350 rpm for 8-12 h at 65 ^{o}C, the reaction mixture was cooled to room temperature, filtered using a 20 mL syringe having a frit and washed with copious amount of solvents DMF (3 x 20 ml), MeOH (3 x 20 ml) and DCM (5 x 20 ml), dried under high vacuum for 8 h and used for further reactions.

Synthesis of compound 5: For the parallel synthesis of each member of library, the resin 4 (200 mg) was placed in a vial, DCM 3 mL was added for pre-swelling. To the pre-swelled resin, pyridine (1.0 mL) and commercially available acid chlorides (125 mg) were added and subjected to shaking at 330-350 rpm for 1 h at the room temperature, the resin was then filtered using a 20 mL syringe having a frit and washed with copious amount of solvents DMF (3 x 20 ml), MeOH (3 x 20 ml) and DCM (5 x 20 ml), dried under high vacuum for 8 h and used for further reactions.

Synthesis of compound 6 (cleavage protocol): To the resin 5 (200 mg), the cleavage solution 2% TFA in DCM (5 ml) solution was added and incubated for 30 min at room temperature. The reaction mixture was filtered using a 20 mL syringe having a frit under vacuum and was washed twice with DCM (10 ml). The filtrate was then neutralized using 2.5% aqueous ammonia in acetonitrile (10 ml approximately). The solvents were evaporated and the crude product was subjected to purification using preparatory HPLC to get the pure compound as dark brown solid and purity was analyzed by LC/MS instrument. LC/MS gradient condition was 5 % ACN to 100 % ACN in water with 4.3 x 50 mm C18 column. All HPLC solvent contains 0.1% formic acid for the LC/MS analysis.

Synthesis of compound 7; Chloroacetylation of AD: To the compound 6 (1.0 equiv) in acetonitrile (2.5 mL), 3.0 mL of saturated $NaHCO_3$ solution was added followed by chloroacetyl chloride (2.5 equiv) at 0 ^oC. The reaction was monitored by LC-MS upon completion of the reaction, ethyl acetate (25.0 mL) was added and layers were separated. The organic layer was washed twice with saturated $NaHCO_3$ solution (10 mL) and the organic layer was dried over sodium sulphate, evaporated to get pure product.

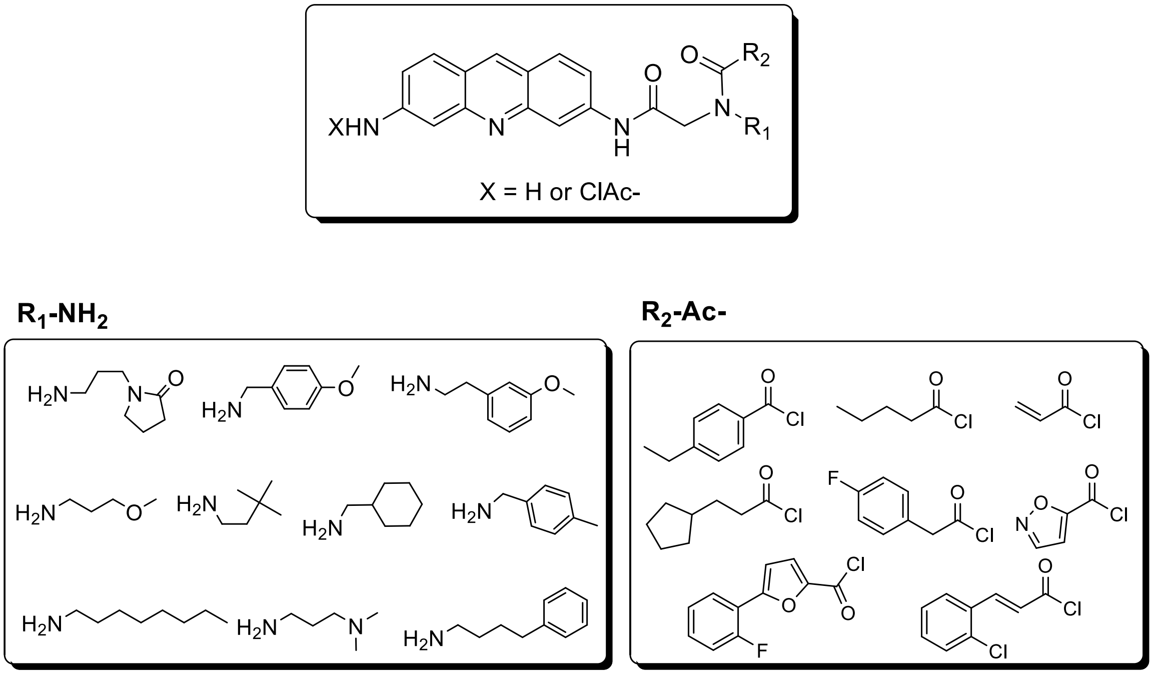

Supplementary Figure 24. General structure of the AD and ADCA library. Upper panel indicates the backbone structure of the compound library (X = H for AD; ClAc- for ADCA). Low panels indicate the R1 and R2 region building blocks for generating library compound. All the building blocks are from commercially available primary amines and acid chlorides

1. Characterization of CDg16 (AD-H9, AD{49, 396})

: CDg16 was prepared by general AD library synthesis protocol and characterized by ^{1}H-NMR, ^{13}C-NMR, High Resolution Mass, Low resolution LC-MS and absorption/emission spectroscopy.

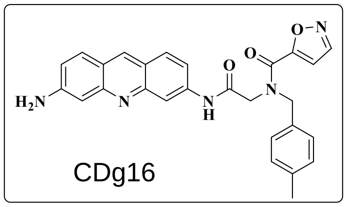

Brown solid. ^{1}H NMR (500 MHz, DMSO) δ 10.41 (s, 2H), 8.73 (dd, J = 25.5, 1.6 Hz, 2H), 8.63 (d, J = 3.7 Hz, 2H), 8.27 (d, J = 24.5 Hz, 2H), 8.18 (s, 2H), 7.92 (dd, J = 8.7, 6.6 Hz, 2H), 7.80 (d, J = 8.9 Hz, 2H), 7.44 (dd, J = 34.0, 8.9 Hz, 2H), 7.29 (d, J = 7.8 Hz, 2H), 7.21 (m, 4H), 7.05 (dd, J = 9.0, 1.4 Hz, 2H), 6.98 (dd, J = 13.2, 1.6 Hz, 2H), 6.87 (s, 2H), 6.27 (s, 4H), 4.77 (s, 2H), 4.71 (s, 2H), 4.38 (s, 2H), 4.26 (s, 2H), 2.30 (s, 3H), 2.28 (S, 3H). (The compound exits as tautomers). At 350K (Variable Temperature) ^{1}H NMR (500 MHz, DMSO) δ 10.16 (s, 1H), 8.65 (s, 1H), 8.60 (s, 1H), 8.22 (s, 1H), 8.20 – 8.13 (m, 1H), 7.90 (d, J = 9.0 Hz, 1H), 7.79 (d, J = 9.0 Hz, 1H), 7.46 (s, 1H), 7.26 (s, 2H), 7.20 (d, J = 7.8 Hz, 2H), 7.07 (dd, J = 8.9, 1.8 Hz, 2H), 6.96 – 6.88 (m, 2H), 4.76 (s, 2H), 4.33 (s, 2H), 2.30 (S, 3H). ^{13}C NMR (126 MHz, DMSO) δ 167.7, 167.1, 163.8, 162.5, 159.4, 159.3, 154.4, 151.6, 151.5, 148.5, 142.4, 141.8, 137.8, 137.2, 133.6, 131.2, 130.5, 129.8, 129.4, 128.7, 127.7, 125.8, 120.9, 120.4, 120.3, 118.3, 107.5, 53.1, 51.6, 50.5, 50.0, 21.5, 21.1. HRMS calcd for $C_{27}H_{24}N_5O_3$ [M+H] 466.1874; Found: 466.1878.

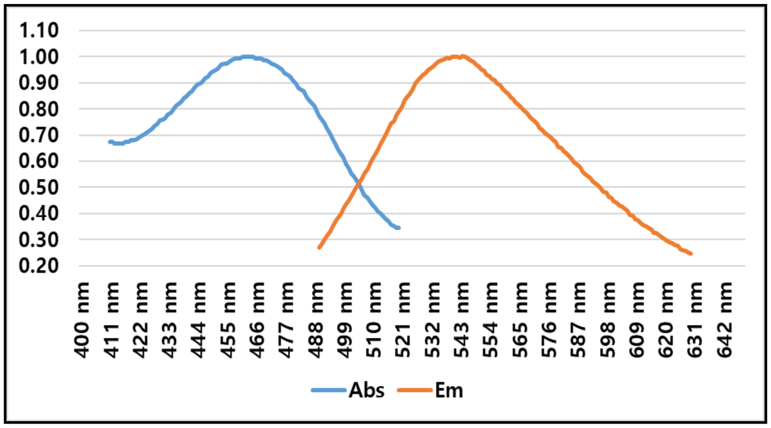

Supplementary Figure 25. Normalized absorption and emission spectroscopic property of CDg16. X- and y- axis represent wavelength and relative intensity, respectively. Abs, absorption; Em, emission spectrum.

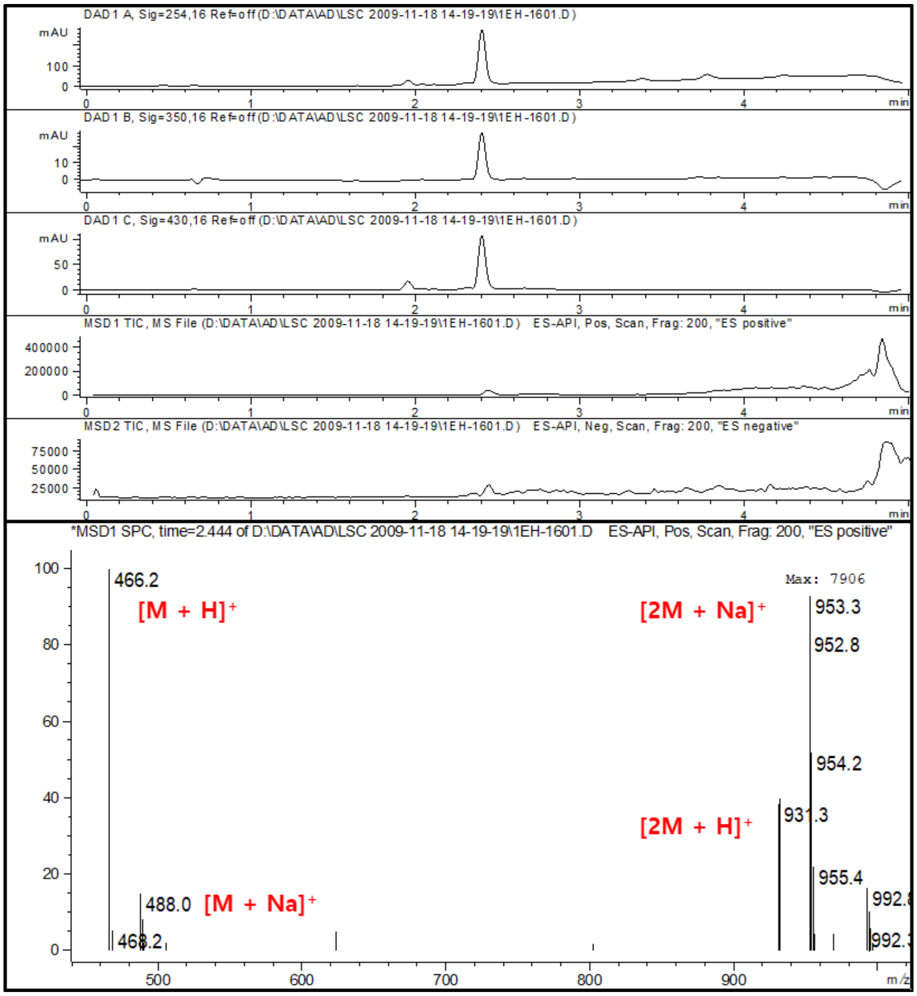

Supplementary Figure 26. LC/MS Chromatogram of CDg16. Liquid chromatography (upper panel) and mass spectrometry (lower panel) for CDg16 were presented. Multiple ionization patterns are indicated in red.

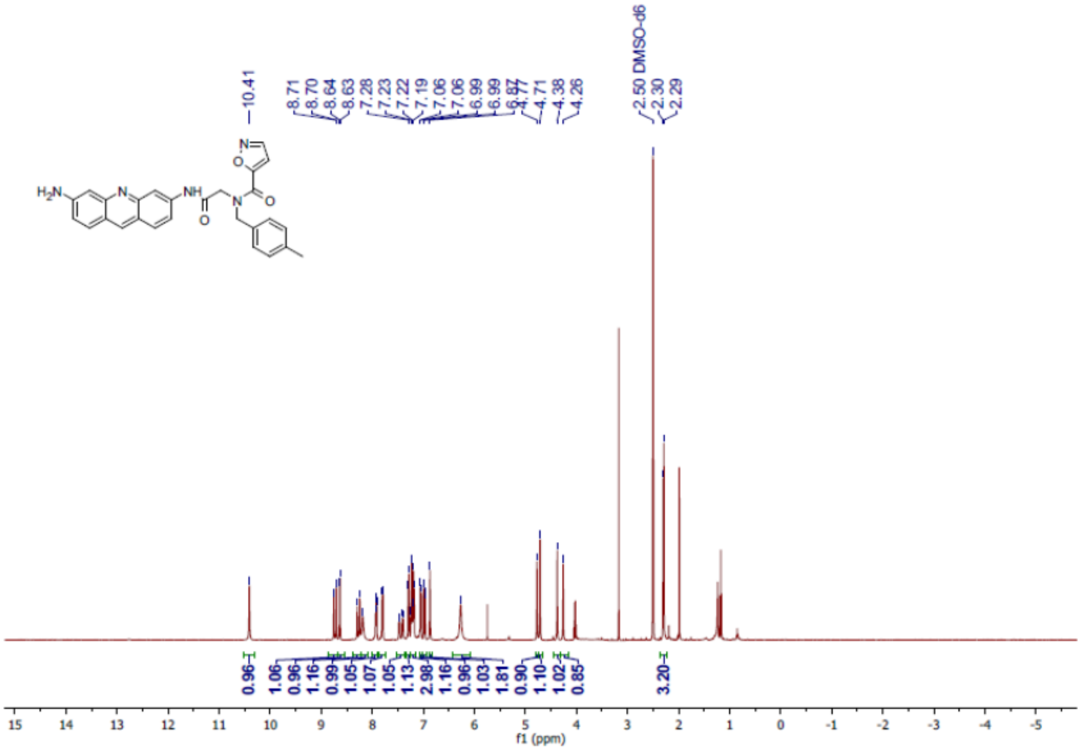

Supplementary Figure 27. ^{1}H-NMR of the compound CDg16 (500 MHz, DMSO-d6).

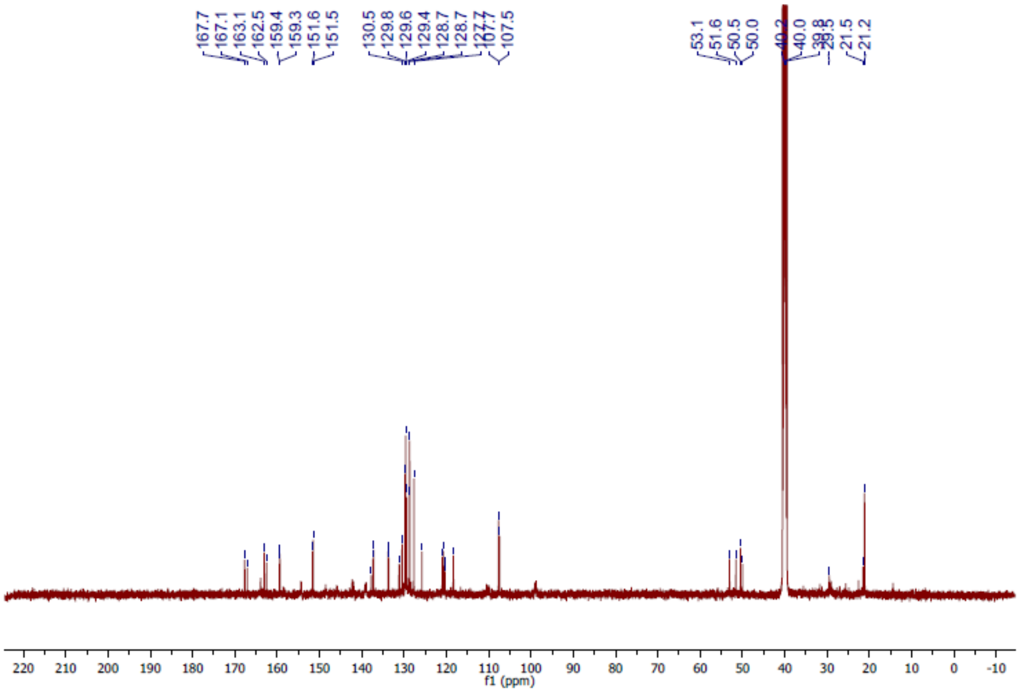

Supplementary Figure 28. ^{13}C-NMR of the compound CDg16 (126 MHz, DMSO-d6).

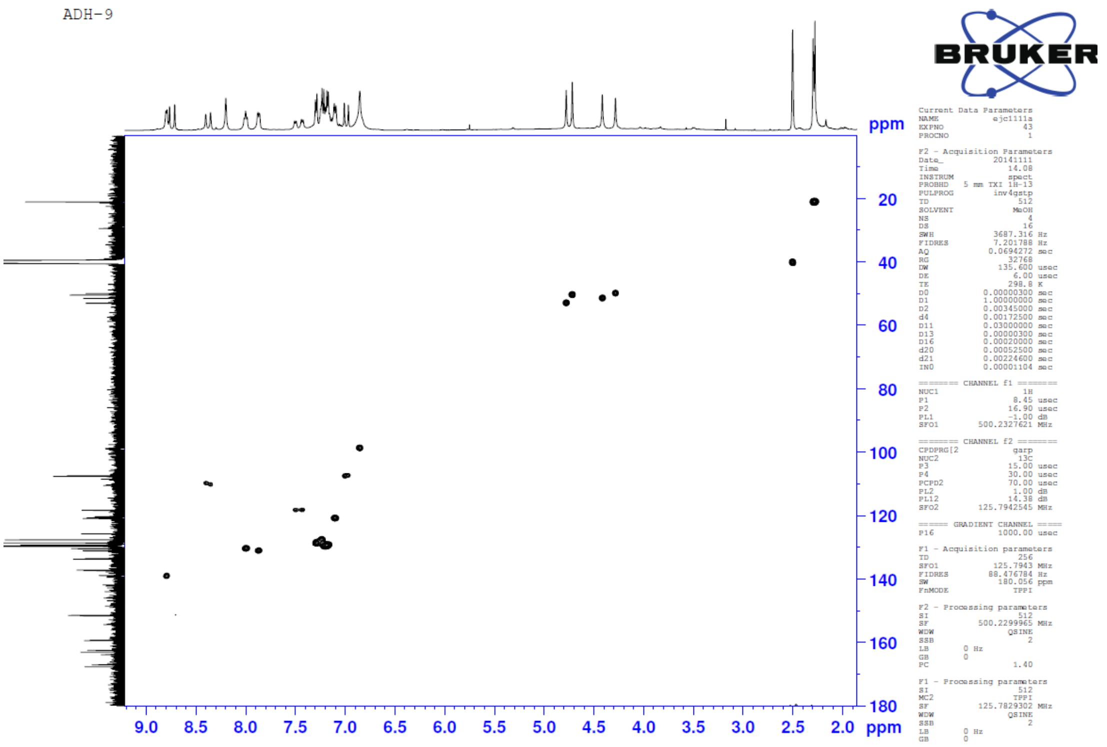

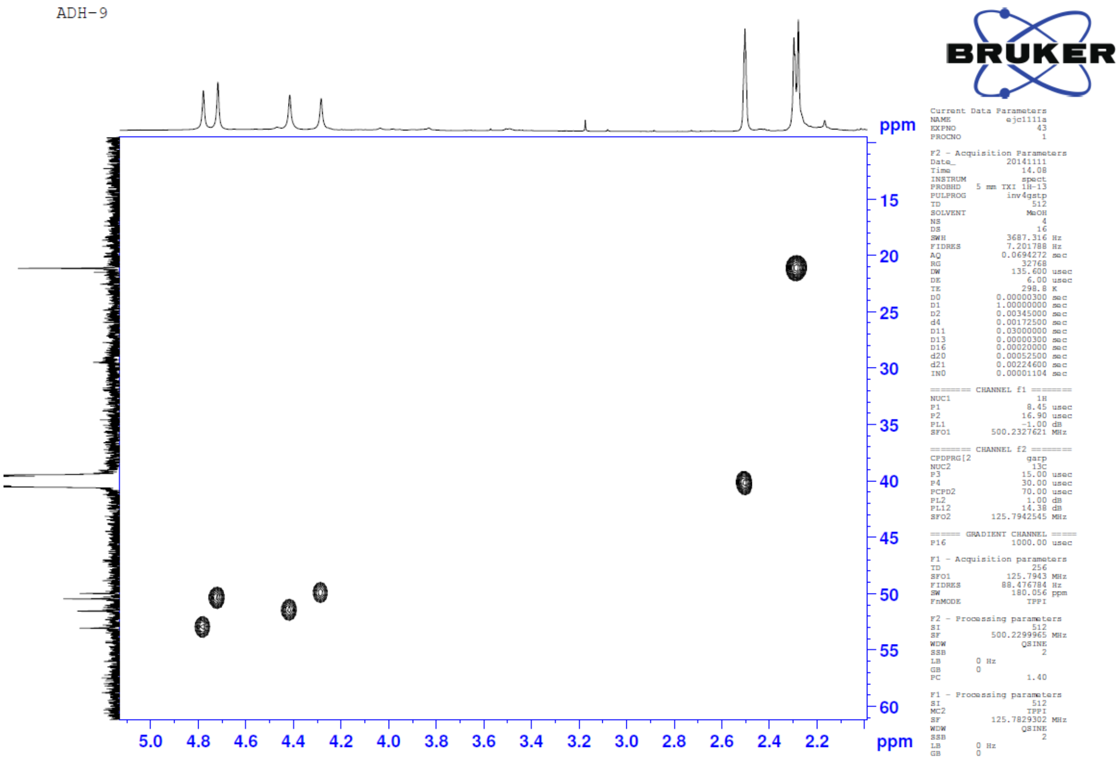

Supplementary Figure 29. ^{1}H-^{13}C-COSY of the compound CDg16 (DMSO-d6).

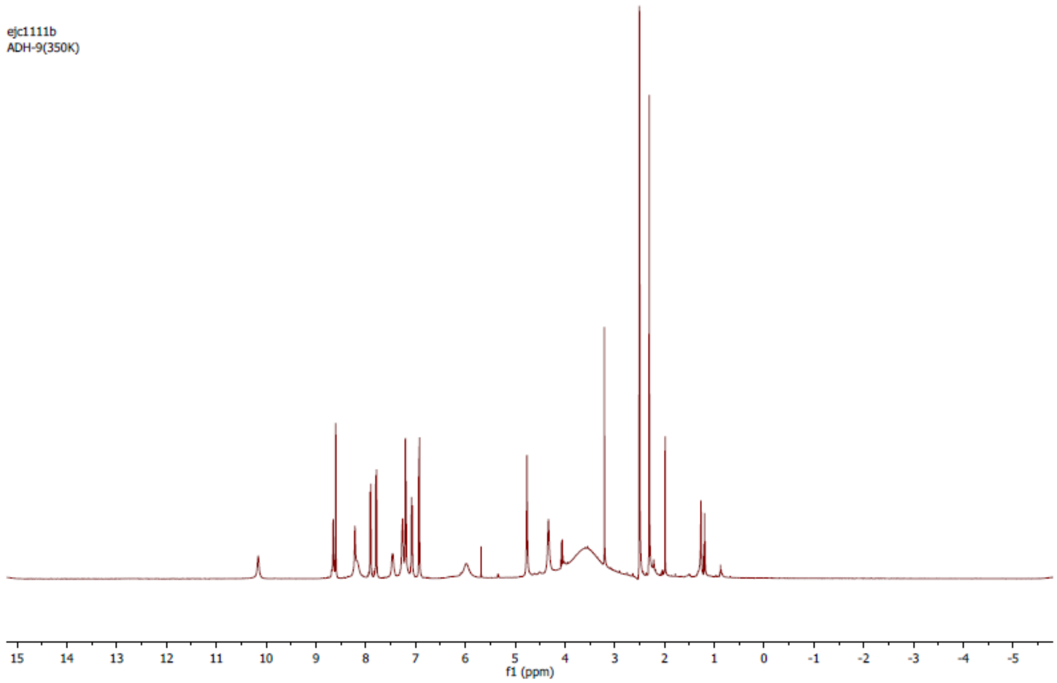

Supplementary Figure 30. Variable temperature ^{1}H-NMR (350 K) of the compound CDg16 (DMSO-d6, 500 MHz).

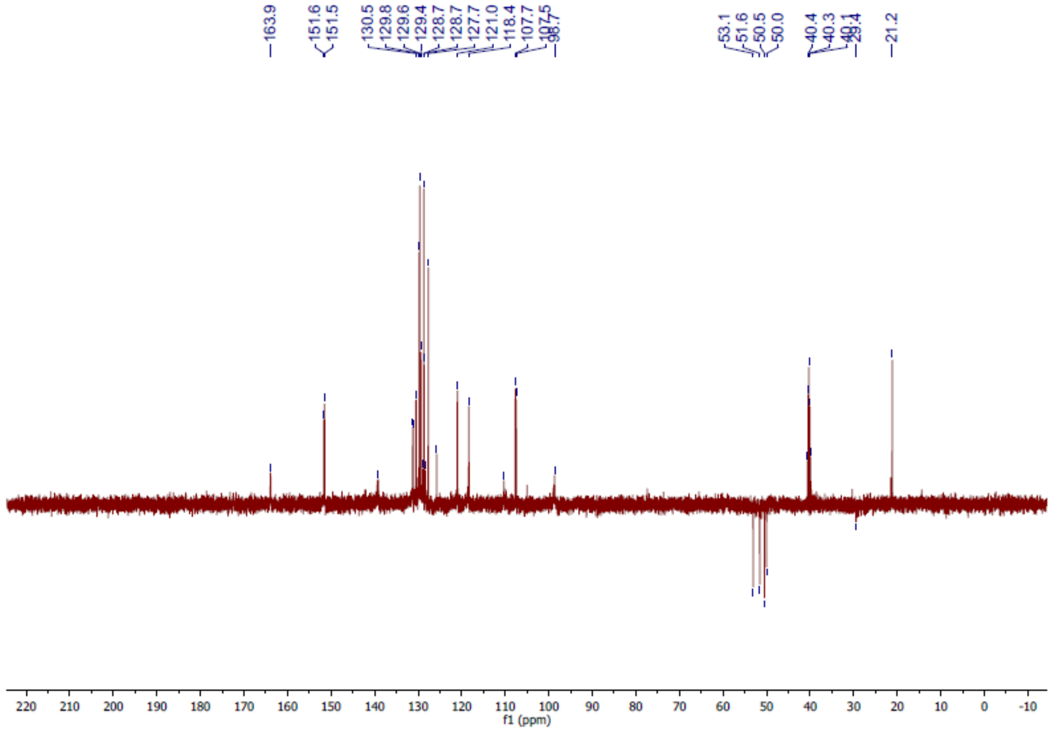

Supplementary Figure 31. DEPT (135) of the compound CDg16 (DMSO-d6).

Mass Spectrum SmartFormula Report

Analysis Info

Analysis Name	D:\Data\Chemistry\2014 Sample\Oct 2014\1031-E\ADH9-1.d	Acquisition Date	10/31/2014 6:19:00 PM
Method	YCH_Pos-150-1800.m	Operator	default user
Sample Name	ADH9	Instrument / Ser#	micrOTOF-Q II 10269
Comment	Prof. Chang Young-Tae		

Acquisition Parameter

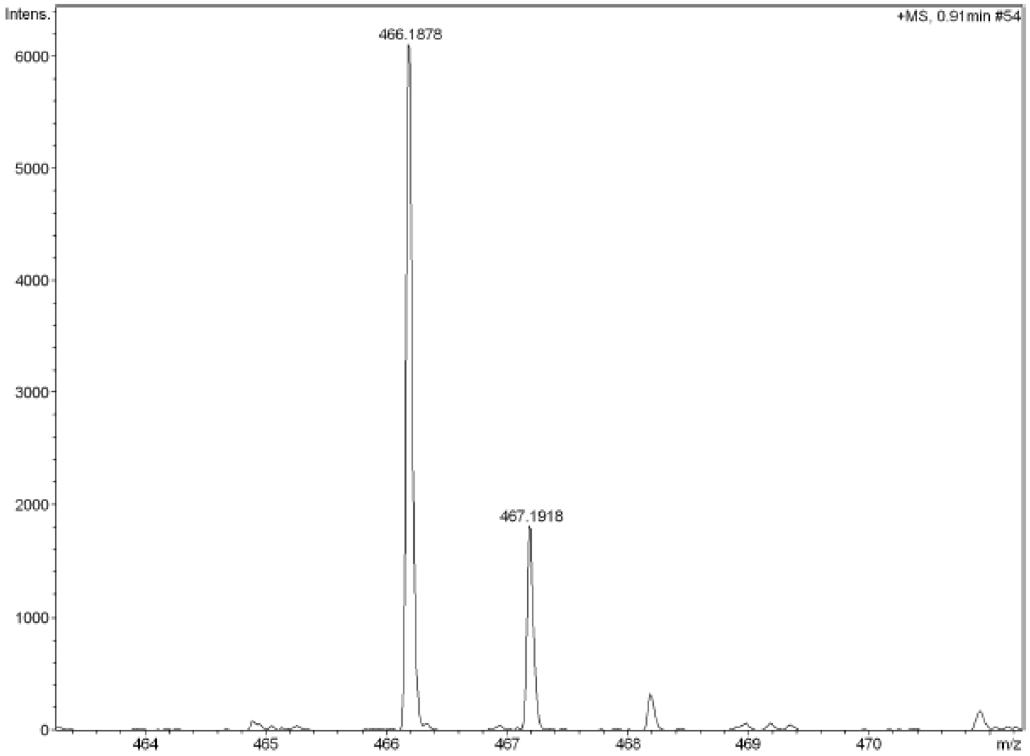

Supplementary Figure 32. High Resolution Mass Spectrum of the compound CDg16.

2. Structure coding and spectroscopic property summary of AD, ADCA library

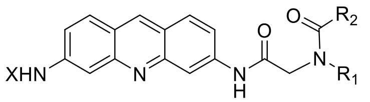

X = H or ClAc-

		R1	M11	M92	M102	M131	M165	M381	M382	M396	M384	M427
			2	3	4	5	6	7	8	9	10	11
R2			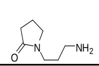	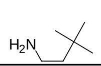	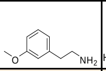	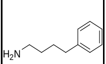	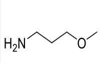	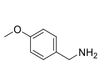	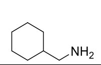	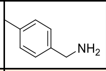	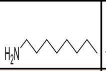	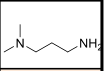
C01	a	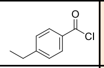	{1,11}	{1,92}	{1,102}	{1,131}	{1,165}	{1,381}	{1,382}	{1,396}	{1,384}	{1,427}
C06	b	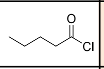	{6,11}	{6,92}	{6,102}	{6,131}	{6,165}	{6,381}	{6,382}	{6,396}	{6,384}	{6,427}
C07	c	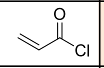	{7,11}	{7,92}	{7,102}	{7,131}	{7,165}	{7,381}	{7,382}	{7,396}	{7,384}	{7,427}
C25	d	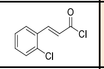	{25,11}	{25,92}	{25,102}	{25,131}	{25,165}	{25,381}	{25,382}	{25,396}	{25,384}	{25,427}
C27	e	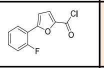	{27,11}	{27,92}	{27,102}	{27,131}	{27,165}	{27,381}	{27,382}	{27,396}	{27,384}	{27,427}
C29	f	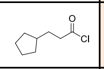	{29,11}	{29,92}	{29,102}	{29,131}	{29,165}	{29,381}	{29,382}	{29,396}	{29,384}	{29,427}
C36	g	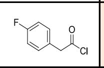	{36,11}	{36,92}	{36,102}	{36,131}	{36,165}	{36,381}	{36,382}	{36,396}	{36,384}	{36,427}
C49	h	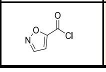	{49,11}	{49,92}	{49,102}	{49,131}	{49,165}	{49,381}	{49,382}	{49,396}	{49,384}	{49,427}

Supplementary Figure 33. The codes for the AD and ADCA library compounds.
