# Supplementary material for: Imaging inflammation using an activated macrophage probe with Slc18b1 as the activation-selective gating target
Source: Nat Commun. 2019 Mar 7;10:1111. doi: 10.1038/s41467-019-08990-9 (PMC6405920; doi:10.1038/s41467-019-08990-9)
Supplement: Supplementary file 3 — Description of Additional Supplementary Files [file 41467_2019_8990_MOESM3_ESM.docx]

**Description of Additional Supplementary Files**

File Name: Supplementary Movie 1

Description: Real-time tracking of activating macrophages with CDg16. Raw264.7 cells were incubated with lipopolysaccharide (LPS, 100 ng/mL), interferon-gamma (IFNγ, 20 ng/mL) and CDg16 (1 µM) at the same time. For 36 hours, the behaviors of activating macrophages and CDg16 responses were observed at the same position. CDg16 staining cells appeared in 8 hours after LPS and IFNγ treatment.

File Name: Supplementary Data 1

Description: Spectroscopic properties of the AD and ADCA library. The molecular weight (M.W.), absorbance, emission wavelength, extinction coefficiency (Ext. coeff) and quantum yield (QY) properties of 160 library compounds are listed.

File Name: Supplementary Data 2

Description: Human SLC gene list used for CRISPR activation. Information of the 380 human SLC genes used for developing a systemic screening approach. GeneID, Entrez gene ID.

File Name: Supplementary Data 3

Description: sgRNA Sequences in the SLC-CRISPRa pools. Information of the 3,800 sgRNA sequences for targeting 380 human SLC genes were listed. tss, transcription start site.

File Name: Supplementary Data 4

Description: LC/MS data of AD library. LC/MS data showing the purity and molecular weight of the AD library compounds. Each compound code is corresponding to the information of Supplementary Figure 33.
