# Supplementary material for: Imaging inflammation using an activated macrophage probe with Slc18b1 as the activation-selective gating target
Source: Nat Commun. 2019 Mar 7;10:1111. doi: 10.1038/s41467-019-08990-9 (PMC6405920; doi:10.1038/s41467-019-08990-9)
Supplement: Supplementary file 8 — Supplementary Data 4 [file 41467_2019_8990_MOESM8_ESM.doc]

**Representative LC-MS chromatogram**.

**C01M11**

**C06M11**

**C07M11**

**C25M11**

**C27M11**

**C29M11**

**C36M11**

**C49M11**

**C01M92**

**C06M92**

**C07M92**

**C25M92**

**C27M92**

**C29M92**

**C36M92**

**C49M92**

**C01M102**

**C07M102**

**C25M102**

**C27M102**

**C29M102**

**C36M102**

**C49M102**

**C01M131**

**C06M131**

**C07M131**

**C25M131**

**C27M131**

**C29M131**

**C36M131**

**C43M131**

**C01M165**

**C06M165**

**C07M165**

**C25M165**

**C27M165**

**C29M165**

**C36M165**

**C49M165**

**C01M381**

**C06M381**

**C07M381**

**C27M381**

**C29M381**

**C36M381**

**C49M381**

**C01M382**

**C06M382**

**C07M382**

**C28M382**

**C27M382**

**C29M382**

**C36M382**

**C49M382**

**C01M396**

**C06M396**

**C07M396**

**C25M396**

**C27M396**

**C29M396**

**C36M396**

**C49M396**

**C01M384**

**C06M384**

**C07M384**

**C25M384**

**C27M384**

**C29M384**

**C36M384**

**C49M384**

**C01M427**

**C06M427**

**C07M427**

**C25M427**

**C27M427**

**C29M427**

**C36M427**

**C49M427**
