# Supplementary material for: Imaging inflammation using an activated macrophage probe with Slc18b1 as the activation-selective gating target
Source: Nat Commun. 2019 Mar 7;10:1111. doi: 10.1038/s41467-019-08990-9 (PMC6405920; doi:10.1038/s41467-019-08990-9)
Supplement: Supplementary file 9 — Reporting Summary [file 41467_2019_8990_MOESM9_ESM.pdf]

nature research

Corresponding author(s): Jin-Soo Kim and Young-Tae Chang

Last updated by author(s): Jan 31, 2019

Reporting Summary

Nature Research wishes to improve the reproducibility of the work that we publish. This form provides structure for consistency and transparency in reporting. For further information on Nature Research policies, see Authors & Referees and the Editorial Policy Checklist.

Statistics

For all statistical analyses, confirm that the following items are present in the figure legend, table legend, main text, or Methods section.

n/a	Confirmed	
☐	☒	The exact sample size (*n*) for each experimental group/condition, given as a discrete number and unit of measurement
☐	☒	A statement on whether measurements were taken from distinct samples or whether the same sample was measured repeatedly
☐	☒	The statistical test(s) used AND whether they are one- or two-sided<br>*Only common tests should be described solely by name; describe more complex techniques in the Methods section.*
☒	☐	A description of all covariates tested
☒	☐	A description of any assumptions or corrections, such as tests of normality and adjustment for multiple comparisons
☐	☒	A full description of the statistical parameters including central tendency (e.g. means) or other basic estimates (e.g. regression coefficient) AND variation (e.g. standard deviation) or associated estimates of uncertainty (e.g. confidence intervals)
☐	☒	For null hypothesis testing, the test statistic (e.g. *F*, *t*, *r*) with confidence intervals, effect sizes, degrees of freedom and *P* value noted<br>*Give P values as exact values whenever suitable.*
☒	☐	For Bayesian analysis, information on the choice of priors and Markov chain Monte Carlo settings
☒	☐	For hierarchical and complex designs, identification of the appropriate level for tests and full reporting of outcomes
☒	☐	Estimates of effect sizes (e.g. Cohen's *d*, Pearson's *r*), indicating how they were calculated

Our web collection on statistics for biologists contains articles on many of the points above.

Software and code

Policy information about availability of computer code

Data collection	MetaXpress (Molecular Devices), Harmony 4.5 (Perkin Elmer), NIS-Elements Basic Research (Nikon)
Data analysis	ImageJ, MetaXpress (Molecular Devices), Harmony 4.5 (Perkin Elmer), NIS-Elements Basic Research (Nikon) and Flowjo 10.2 (TreeStar) were used to analyze the data.

For manuscripts utilizing custom algorithms or software that are central to the research but not yet described in published literature, software must be made available to editors/reviewers. We strongly encourage code deposition in a community repository (e.g. GitHub). See the Nature Research guidelines for submitting code & software for further information.

Data

Policy information about availability of data

All manuscripts must include a data availability statement. This statement should provide the following information, where applicable:

- Accession codes, unique identifiers, or web links for publicly available datasets
- A list of figures that have associated raw data
- A description of any restrictions on data availability

Any supplementary information and videos are available in the online version of the paper. The deep sequencing data that support the findings of this study have been uploaded to the NCBI Sequence Read Archive under Bioproject accession code PRJNA516962. All other data are available from the authors upon request.

Field-specific reporting

Please select the one below that is the best fit for your research. If you are not sure, read the appropriate sections before making your selection.

☒ Life sciences ☐ Behavioural & social sciences ☐ Ecological, evolutionary & environmental sciences

For a reference copy of the document with all sections, see nature.com/documents/nr-reporting-summary-flat.pdf

Life sciences study design

All studies must disclose on these points even when the disclosure is negative.

Sample size	1. For screening, at least three independent experiments was performed to select reproducible probes to Raw264.7 and THP-1 cell lines.<br>2. All the images and flow cytometry from primary macrophages/tissues/ex vivo samples were from at least three independent experiments for reproducibility.<br>3. Immunolabeling data of supplementary information are from at least three independent experiments.<br>4. All of the in vitro/ex vivo images were from at least three independent experiments for reproducibility.
Data exclusions	No exclusion data.
Replication	All attempts at replication were successful.
Randomization	Mouse samples were collected from wild-type or ApoE knock-out transgenic mice (The Jackson Laboratory). All the sample allocations were random.
Blinding	All the data collection and analysis were from blinded with randomized samples.

Reporting for specific materials, systems and methods

We require information from authors about some types of materials, experimental systems and methods used in many studies. Here, indicate whether each material, system or method listed is relevant to your study. If you are not sure if a list item applies to your research, read the appropriate section before selecting a response.

Materials & experimental systems

n/a	Involved in the study	
☐	☒	Antibodies
☐	☒	Eukaryotic cell lines
☒	☐	Palaeontology
☐	☒	Animals and other organisms
☒	☐	Human research participants
☒	☐	Clinical data

Methods

n/a	Involved in the study	
☒	☐	ChIP-seq
☐	☒	Flow cytometry
☒	☐	MRI-based neuroimaging

Antibodies

Antibodies used	Rat anti-mouse CD86 (BD Pharmigen, 553689), AlexaFluor 647 conjugated goat anti-rat IgG (ThermoFisher Scientific, A-21247), AlexaFluor 594 conjugated anti-B7-2/CD86 antibody (R&D Systems, FAB741T), AlexaFluor 647 conjugated anti-iNOS antibody (Abcam, ab209027), APC conjugated anti-CD206 (MMR) antibody (BioLegend, 141708), PE/Cy7 conjugated anti-CD38 (BioLegend, 102717), PerCP/Cy5.5 conjugated anti-CD45 antibody (BioLegend, 147706), AlexaFluor 594 conjugated anti-human CD86 (R&D Systems, FAB141T) and AlexaFluor 594 conjugated anti-human CD206 (BioLegend, 321116), CD45-PerCP-Cy5.5 (Biolegend, 147706), CD86-Alexa Fluor 594 (R&D systems, FAB741T), CD38-PE-Cy7 (Biolegend, 102717), CD206-APC (Biolegend, 141708).
Validation	The companies provided the validation information in their websites.

Eukaryotic cell lines

Policy information about cell lines

Cell line source(s)	All cell lines were purchased from ATCC.
Authentication	Raw264.7 was authenticated by its morphology and growth rate under normal culture condition and by its expression of CD86 and morphological changes in response to LPS/IFNg. THP-1 was authenticated by its attachment to culture dish after differentiation to macrophage and by its morphological changes in response to LPS/IFNg.

Mycoplasma contamination	Not analyzed. The cell lines were directly purchased from ATCC, and used within 30 passages.
Commonly misidentified lines (See ICLAC register)	N/A

Animals and other organisms

Policy information about studies involving animals; ARRIVE guidelines recommended for reporting animal research

Laboratory animals	The study used two mouse strains as described below in detail:<br>1. C57BL/6J mice for control and acute inflammation model, both gender, 18 week to 23 week ages.<br>2. ApoE Knock-out mice for atherosclerosis model, both gender, 18 week to 23 week ages
Wild animals	N/A
Field-collected samples	N/A
Ethics oversight	All animal experimental procedures were performed in accordance with a protocol approved by the Institutional Animal Care and Use Committee for Biological Resource Center at A*STAR, Singapore (IACUC #151032 and #151033).

Note that full information on the approval of the study protocol must also be provided in the manuscript.

Flow Cytometry

Plots

Confirm that:

☒ The axis labels state the marker and fluorochrome used (e.g. CD4-FITC).

☒ The axis scales are clearly visible. Include numbers along axes only for bottom left plot of group (a 'group' is an analysis of identical markers).

☒ All plots are contour plots with outliers or pseudocolor plots.

☒ A numerical value for number of cells or percentage (with statistics) is provided.

Methodology

Sample preparation	Raw264.7, CRISPR Knock-out Raw264.7 and SLC-CRISPRa Knock-in HeLa cells were disociated with Accutase/DNase I mixture.<br>Isolated cells were incubated with CDg16 under normal culture media.<br>Flow cytometry or FACS experiments were performed without washing the probes unless otherwise indicated.
Instrument	Flow cytometry and FACS was performed using Fortessa (BD Biosciences) and MoFlo XDP (ML99030, Beckman Coulter), respectively.
Software	Data analysis was performed with FlowJo 10.2 software (TreeStar).
Cell population abundance	Not applicable as the CDg16-derived fluorescence signal was largely decreased after sorting.
Gating strategy	The live cell populations selected with the live FSC/SSC gating (high FSC values used for sorting live cells, 83 –90% of Hela and 25-50% of activated Raw264.7 cells were positive from the total events). The live cells were sub-gated with FSC-A/FSC-H to discriminate single cell population from aggregates (Singlets, 89-98% of the parental populations). The singlets were used for flow cytometry or FACS based on the CDg16 signal intensity.

☒ Tick this box to confirm that a figure exemplifying the gating strategy is provided in the Supplementary Information.
